# Misfit Layered Compounds: Insights into Chemical, Kinetic, and Thermodynamic Stability of Nanophases

**DOI:** 10.1021/acs.accounts.4c00412

**Published:** 2024-11-04

**Authors:** Azat Khadiev, M. B. Sreedhara, Simon Hettler, Dmitri Novikov, Raul Arenal, Reshef Tenne

**Affiliations:** ¶ Deutsches Elektronen-Synchrotron DESY, Notkestr. 85, 22607 Hamburg, Germany; ‡ Solid State and Structural Chemistry Unit, Indian Institute of Science, Bengaluru, 560012 India; § Instituto de Nanociencia y Materiales de Aragon (INMA), CSIC-Universidad de Zaragoza, 50018 Zaragoza, Spain; ∥ Laboratorio de Microscopias Avanzadas (LMA), Universidad de Zaragoza, 50018 Zaragoza, Spain; ⊥ ARAID Foundation, 50018 Zaragoza, Spain; # Department of Molecular Chemistry and Materials Science, Weizmann Institute of Science, Rehovot 7610001, Israel

## Abstract

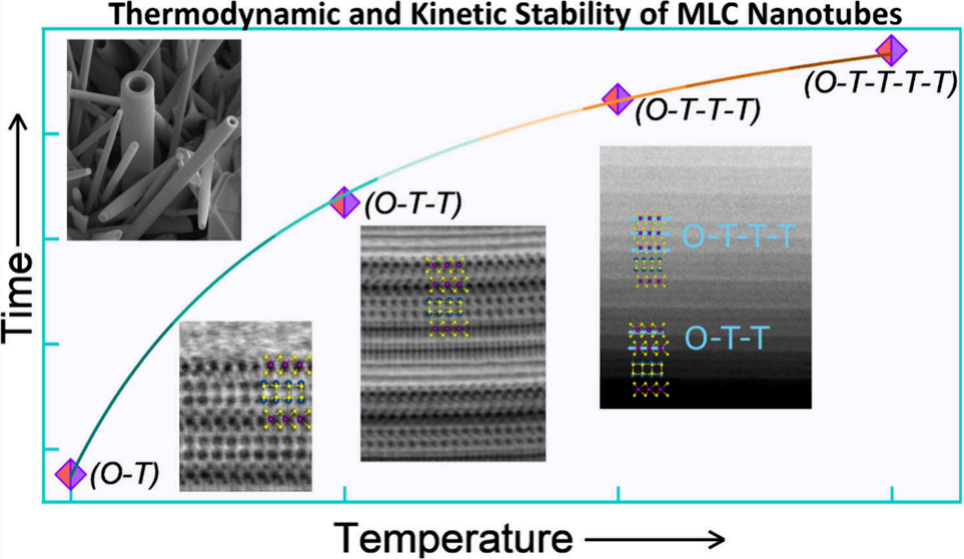

Compounds with layered structures (2D-materials), like transition metal-dichalcogenides (e.g., MoS_2_), attracted a great deal of interest in the scientific community in recent years. This interest can be attributed to their unique lamellar structure, which induces large anisotropy in their physicochemical properties. Furthermore, owing to the weak van der Waals interaction between the layers, they can be cleaved along the *a–b* plane, which allows fabricating single layers with physical properties entirely different from the bulk material. Moreover, stacking layers of different 2D-materials on top of each other has led to a wealth of new observations, for instance, by twisting two layers with respect to each other and producing Moiré lattice. Another outstanding property of inorganic layer compounds is their tendency to form nanotubes, reported first (for WS_2_) many years ago and subsequently from many other layered compounds.

Among the 2D-materials, misfit layer compounds make a special class with an incommensurate and nonstoichiometric lattice made of an alternating layer with rocksalt structure, like LaS (*O*) and a layer with hexagonal structure, like TaS_2_ (*T*). The lack of lattice commensuration between the two slabs leads to a built-in strain, which can be relaxed via bending. Consequently, nanotubes have been produced from numerous MLC compounds over the past decade and their structure was elucidated.

Owing to their large surface area, nanostructures are generally metastable and tend to recrystallize into microscopic crystallites via different mechanisms, like Ostwald ripening, or chemically decompose and then recrystallize. The stability of nanostructures at elevated temperatures has been investigated quite scarcely so far. In this perspective, electron microscopy as well as synchrotron-based X-ray absorption and reflection techniques were used to elucidate the chemical selectivity and decomposition routes of rare-earth based MLC nanotubes prepared at elevated temperatures (800–1200 °C).

As for the chemical selectivity, entropic effects are expected to dictate the random distribution of the chalcogen atoms on the anion sites of the MLC nanotubes at elevated temperatures. Nonetheless, the sulfur atoms were found to bind exclusively to the rare-earth atom (Ln = La, Sm) of the rocksalt slab and the selenium to the tantalum of the hexagonal TX_2_ slab. This uncommon selectivity was not found in other kinds of nanotubes like WSe_2*x*_S_2(1–*x*)_. In other series of experiments, the lack of utter symmetry in the multiwall nanotubes leads to exclusions of certain X-ray (0*kl*) reflections, which was used to distinguish them from the bulk crystallites. The transformation of Ln-based MLC nanotubes into microscopic flakes was followed as a function of the synthesis temperature (800–1200 °C) and the synthesis time (1–96 h). Furthermore, sequential high-temperature transformations of the (*O-T*) lattice into (*O-T-T*) and finally (*O-T-T-T*) phases via deintercalation of the LnS slab was observed. This autocatalytic process is reminiscent of the deintercalation of alkali atoms from different layered structure materials. Annealing at higher temperatures and for longer periods of time eventually leads to the decomposition of the ternary MLC into binary metal-sulfide phases, as well as partial oxidation of the product. This study sheds light on the complex mechanism of high-temperature chemical stability of the nanostructures.

## Key References

- SreedharaM. B.; KhadievA.; ZhengK.; HettlerS.; SerraM.; CastelliI. E.; ArenalR.; NovikovD.; TenneR.Nanotubes from Lanthanide-Based Misfit-Layered Compounds: Understanding the Growth, Thermodynamic, and Kinetic Stability Limits. Chem. Mater.2024, 36 ( (9), ), 4736–474938770011 10.1021/acs.chemmater.4c00481PMC11104483.^1^ Demonstrates a synchrotron-based X-ray diffraction approach to study the degradation mechanism of nanophases in bulk powder and gives insights into the degradation mechanism of LnS-TaS_2_ MLC nanotubes at high temperatures.
- SreedharaM. B.; HettlerS.; Kaplan-AshiriI.; RechavK.; FeldmanY.; EnyashinA.; HoubenL.; ArenalR.; TenneR.Asymmetric Misfit Nanotubes: Chemical Affinity Outwits the Entropy at High-temperature Solid-State Reactions. Proc. Natl. Acad. Sci. U.S.A.2021, 118 ( (35), ), e210994511834446565 10.1073/pnas.2109945118PMC8536368.^2^ The study shows that even at elevated temperatures the Ln-based MLC system shows that chemical affinity plays a significant role over the entropy and leads to highly asymmetric nanotubes with broken inversion and time reversal symmetries.
- HettlerS.; SreedharaM. B.; SerraM.; SinhaS. S.; Popovitz-BiroR.; PinkasI.; EnyashinA. N.; TenneR.; ArenalR.YS-TaS_2_ and Y_*x*_La_1–*x*_S-TaS_2_ (0 ≤ *x* ≤ 1) Nanotubes: A Family of Misfit Layered Compounds. ACS Nano2020, 14 ( (5), ), 5445–545832347713 10.1021/acsnano.9b09284PMC7467812.^3^ Explore the detailed structural analysis of misfit nanotubes and the effect of alloying on the yield, stability, and charge transfer characteristics of La(Y)S-TaS_2_ MLC system.

## Introduction

**Figure 1:**
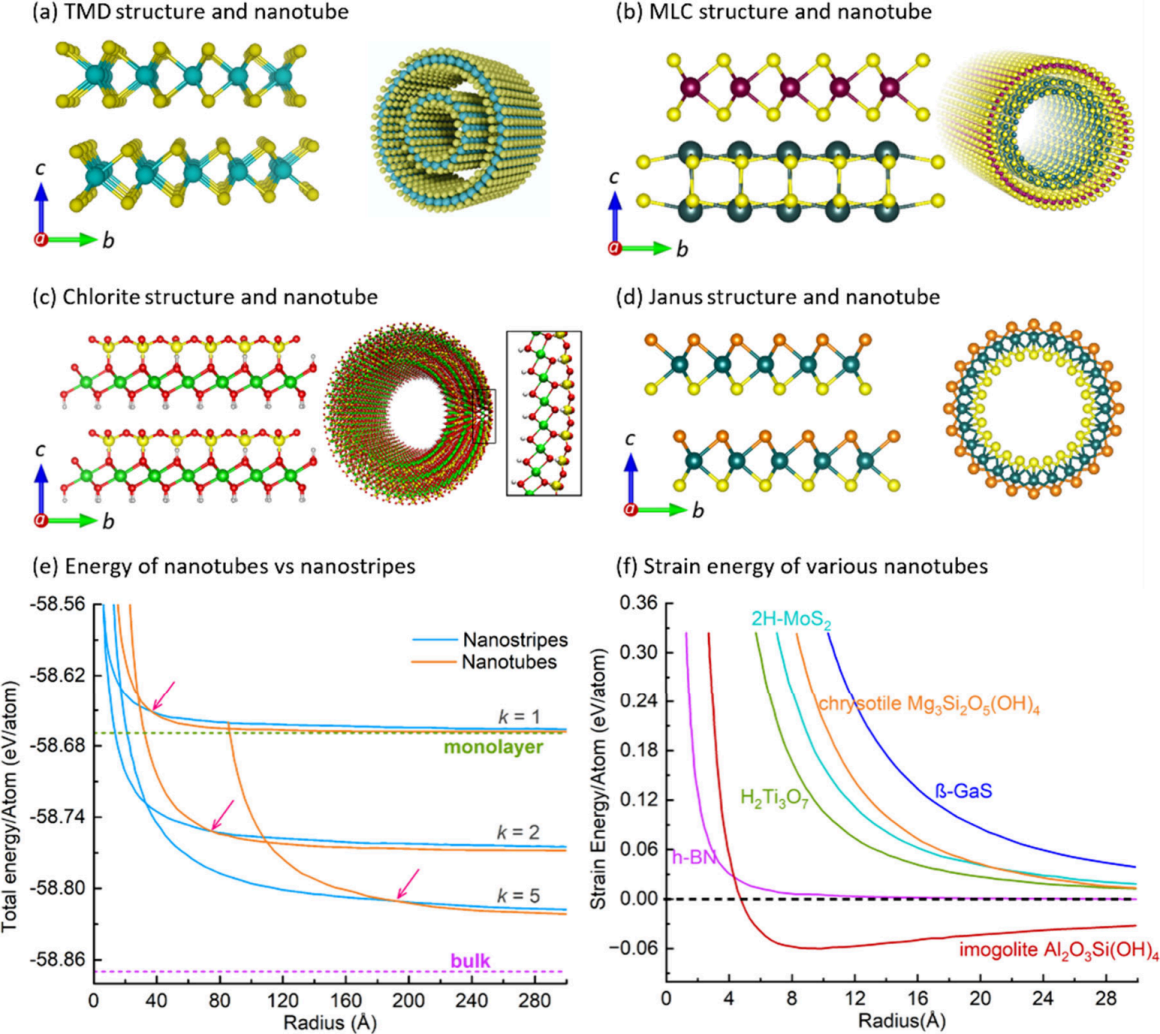
Schematic rendering of the various layered materials (a–d) and their corresponding nanotubes with different strain energy of folding. (a) Structure of TMDCs specifically, MoS_2_/WS_2_ possessing a symmetric layered structure^5^ and a corresponding bilayer nanotube.^25^ (b) Structure of MLCs with alternate stacking of MX and TX_2_ units^24^ and single layer MLC nanotube.^26^ (c) Layered structure of halloysite; the asymmetry between the Al-octahedra and Si-tetrahedra induces high strain forcing folding of the layer.^27−29^ (d) Janus layer with broken inversion symmetry and MoSSe Janus nanotubes.^30^ (e) Stability of the nanotube and nanostripes in comparison with bulk and monolayers. The energy/atom of the materials is reduced with an increasing number of layers stacked together. Note that under a critical size the nanoribbon becomes more stable than the nanotube (crossover point marked by pink arrows).^31^ (f) Total energy of a nanotube calculated for various layered materials as a function of nanotube radius. Note that in contrast to the symmetric compounds, which are metastable (positive strain energy), the minimum in the energy diagram of imogolite signifies its absolute stability compared to the bulk flat structure.^32^

Materials with layered structures, like graphite and transition metal dichalcogenides (TMDCs), have attracted considerable interest in the scientific community for over a century.^4,5^ These compounds are characterized by strong, mostly covalent, bonding between the atoms within the molecular slab (*a–b* plane). The molecular slabs are packed together (along the *c*-axis) via mostly weak van der Waals interlayer interactions. MoS_2_ is one such layered structure material (see Figure 1a), which has been thoroughly investigated in recent years. Compounds with layered structure exhibit highly anisotropic physiochemical properties. One practical aspect of the anisotropy is that they can be easily cleaved in the *a–b* plane.^5^ This practice allows revealing fresh surface suitable for many studies and more recently is a means to exfoliate the crystal and obtain a single layer of that compound. Among this group of anisotropic materials, the family of misfit layered compounds (MLCs) stands-out in its unique structure and physicochemical properties, which are not a simple combination of its binary building blocks. MLCs are incommensurate and nonstoichiometric materials made of periodic stacking of a layer with (distorted) rocksalt structure (MX abbreviated as *O*) alternately with hexagonal TX_2_ (or *T*) layer (Figure 1b). MLC are schematically denoted as (MX)_1+*y*_(TX_2_)_*m*_ (MX-TX_2_ or *O-T* for brevity), where the parameter *m* denotes the number of TX_2_ layers in the repeat unit. In MX-TX_2_ MLCs where M = Sn, Pb, Rare earths; T = Sn, Nb, Ta, and X = S, Se, Te have a common *c*-axis for both subunits, but the (*a–b*) plane of the two layers is incommensurate (usually along the *a*-axis).^6^ In other words, the ratio between the unit cells of the *O* and *T* layers is irrational, which makes MLCs quasi-periodic compounds. The nonstoichiometric composition is expressed through the parameter 1 + *y* = 2*a*_*T*_/*a*_*O*_ (0.08 < *y* < 0.32).

The first misfit compound discussed in the literature was graphite intercalated FeCl_3_.^7^ Although typical MLC materials like PbTiS_3_,^8^ PbNbS_3_,^9^ LnMX_3_^10^ (Ln = rare earth, Bi; M = Ta, Nb, Ti, V; X = S, Se) were synthesized early on, the accurate assignment of their misfit structure was missing. The structure of MLC was revealed first by Kato et al., who studied LaCrS_3_^11^ and more generally by Makovicky and Hyde who also coined the term “misfit” compounds.^6,12−14^ Sinusoidal height modulations studied with scanning probe microscopy are a manifestation of the built-in strain in the incommensurate MLC structure and a hallmark of its lattice relaxation mechanism.^15^ More recently, systematic investigation of the structure of synthetic chalcogenide-based MLC came with the works of Wiegers,^16^ Meerschaut and Rouxel,^17,18^ and later on many others.^19,20^

MLCs have been investigated thoroughly over the years.^16−23^Figure 1b displays an ortho-pseudohexagonal unit of (LaS)_1.13_TaS_2_, along the *c*-direction with overall orthorhombic symmetry and the lattice parameters of TaS_2_ (*a*, *b*= √3·*a*).^24^ In analogy to intercalation compounds, the stability of the MLC structure is augmented by charge transfer from the MX unit to the hexagonal TX_2_. However, in contrast to typical intercalation compounds like Li_*x*_CoO_2_, where the degree of (Li) intercalation (*x*) is variable between zero to one, in the case of pristine MLC, this ratio is constant (1+*y*). In the case of the rare-earth based LnS-TaS_2_ series MLCs are particularly interesting, because most rare earth atoms prefer the +3 valency and hence the stability of these MLC compound is related to a charge transfer from the 5*d* (4f) level of the rare-earth atom to the partially filled 5*d*_*z*^2^_ level of the tantalum atom. Owing to the hyperstoichiometry (1 + *y*) of LnS with respect to TaS_2_ in the MLC, a complete charge transfer from the Ln atom to Ta is not favorable and is partially compensated by defects, antisites, and other kinds of dislocations.

Recently, MLC chemistry received a twist and has been largely expanded by using out-of-equilibrium growth techniques, enabling metastable MLC structures. Most importantly among them is the modulated elemental reactants (MER) technique, which yields turbostratically misaligned MLCs (ferecrystals).^33,34^ In another interesting work,^35^ stable (LaSe)_1.14_NbSe_2_ and (SmS)_1.19_TaS_2_ and also metastable misfit structures, like the sequence LaTa(Se_0.5_S_0.5_)_3_, were prepared by mechanochemical reaction via ball milling and subsequent annealing at 1000 °C.

## Nanotubes

Pauling observed that layered alumino (magnesia)-silicates compounds can be categorized into two families—symmetric and asymmetric ones.^27^ In the asymmetric compounds, see Figure 1c, such as halloysite Al_2_Si_2_O_5_(OH)_4_·2H_2_O) and chrysotile (Mg_3_(Si_2_O_5_)(OH)_4_), the (*a–b*) plane of the alumina (magnesia) octahedra is smaller than that of a pair of the interconnected silica tetrahedra. This asymmetry forces the entire layer to bend. It took another 20 years for such nanotubes to be visually confirmed once transmission electron microscopy (TEM) gained sufficient resolution.^28,29^ A new class of asymmetric 2D-materials, the so-called *Janus* structures, are also notable. Owing to their asymmetric structures, Janus layered compounds are expected to form nanotubes (see Figure 1d). Indeed, Janus nanotubes have been investigated quite thoroughly via *ab initio* methods. For instance, the asymmetric Janus from the TMDCs or BiSI are subdued to a similar driving force for folding into nanotubes.^30^ Such nanotubes exhibit an energy minimum in small diameter and could in principle be a stable phase at ambient conditions.^31,32^ The first MoSSe nanotube synthesized on top of BN nanotube scaffold was recently reported.^36^

More than three decades ago, nanotubes from metal dichalcogenide compounds, like WS_2_^25,37^ (Figure 1a) and h-BN,^38,39^ were reported opening a new chapter in the chemistry and nanotechnology of layered compounds. Unlike the mechanism proposed by Pauling,^27^ here the driving force for nanotubes’ formation requires surmounting of the large elastic energy of folding. The energy gained by healing the dangling bonds on the rim atoms of the slab more than compensates for the folding energy in the given size-range. Since the ratio between rim/bulk atoms increases upon shrinking the length of the slab, the nanotube becomes more stable than the flat 2D nanoflake (having the same number of atoms). This new so-called Kroto–Iijima–Tenne (KIT) mechanism has proven itself over and over again both experimentally and also via numerous *in-silico* calculations.^31,40,41^ However, at a very small radius, the energetic penalty of folding the layer becomes excessively high, making the nanoribbon more stable than the nanotube. This crossover point (red arrow) is clearly shown in Figure 1e. Early *ab initio* calculations showed also^31,40^ that the trilayer WS_2_ (MoS_2_) nanotubes gain extra stability by accommodating larger radii compared to carbon nanotubes (CNT) and also having more than one layer as seen in Figure 1e. Therefore, these multiwall nanotubes are stable over a limited range of diameters, e.g., 10–150 nm. Actually, this figure explains also the great difficulty to synthesize singlewall WS_2_ (MoS_2_) nanotubes, which is so common for CNT. Fundamentally, there is a great difference between the nanotubes obtained via the Pauling mechanism,^27,29,30^ and those produced via the KIT mechanism.^22−25^ While the nanotubes obtained from asymmetric layered compounds, like Janus^21^ are stable, i.e., their strain energy goes through a minimum and is lower than the infinite layer, nanotubes derived from symmetric layered compounds, like CNT, BN, and WS_2_, are metastable and their strain energy is always positive, as demonstrated in Figure 1f. In fact, the global stability of the asymmetric nanotubes could be the reason that imogolite and chrysotile nanotubes are stable on a geological time scale (billions of years) and are mined on the surface of the earth.

**Figure 2:**
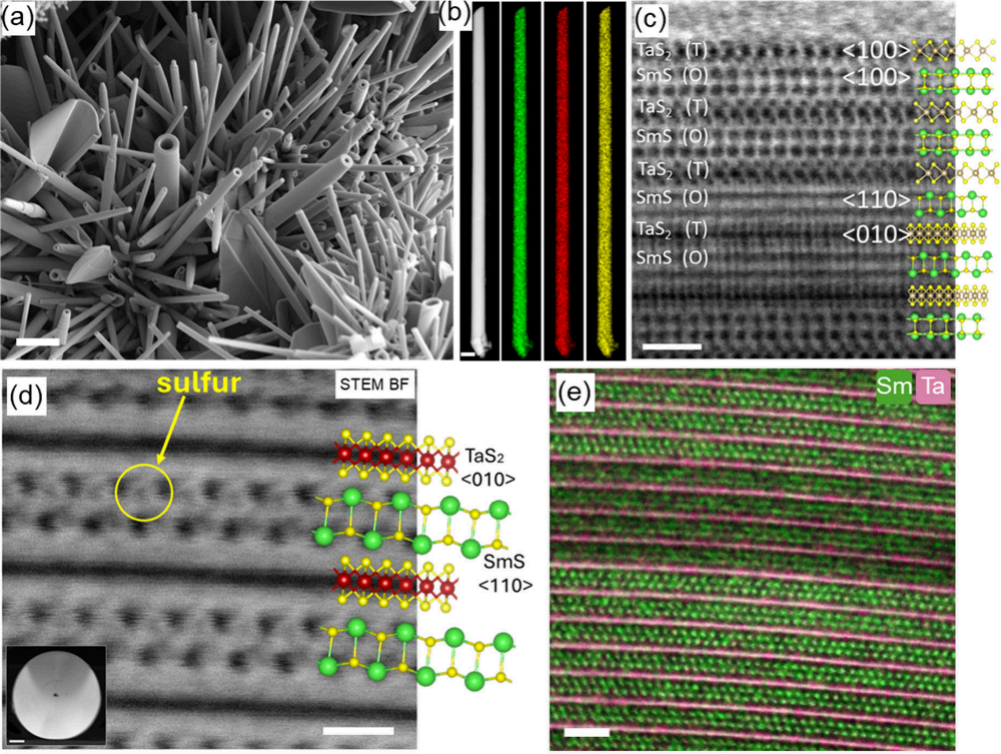
SEM image of SmS-TaS_2_ nanotubes obtained by chemical vapor transport reaction at high temperatures using Cl as transport agent, scale bar 2 μm. (b) STEM-HAADF image of a single SmS-TaS_2_ nanotube and the corresponding EDS chemical maps: Sm, green; Ta,red; and S, yellow; scale bar is 200 nm. (c) HR-STEM image of a nanotube showing the alternate stacking of rocksalt SmS and trigonal prismatic TaS_2_ in the misfit lattice structure. Model structure and crystallographic orientations are overlaid on the STEM image. Scale bar: 1 nm. The contrast difference between first two and adjacent ones correspond to the (*O-T*)(*O-T*)′ hyperperiodicity. (d) Atomic resolution HR-STEM-BF image of a portion of the nanotube lamellae; a low magnification image of the cross-section shown in the inset, scale bar 100 nm. The magnified image reveals the sulfur atoms adjacent to samarium atoms in the rock salt unit, scale bar 1 nm. (e) Atomic resolution STEM-EDS chemical mapping of individual nanotube in cross-section geometry, Ta (red) and Sm (green) layers, scale bar 2 nm (Reproduced with modification from ref (42)., copyright 2024, CC-BY 4.0, Authors).

Presumably, the expedient combination of both the Pauling and the KIT mechanisms permitted successful synthesis of numerous MLC nanotubes (schematically presented in Figure 1b) in tangible amounts.^26^Figures 2a and S1 (in Supporting Information, SI) displays scanning electron microscopy image (SEM) of SmS-TaS_2_ and LaS-TaS_2_ nanotubes mixed with few MLC flakes of the same composition. Figure 2b shows a scanning transmission electron microscopy high-angle annular dark-field (STEM-HAADF) image of an individual nanotube and STEM energy dispersive X-ray spectroscopy (STEM-EDS) mapping of the different atoms making the MLC nanotube. The uniform distribution of the different atoms is testimony to the robustness of the nanotube synthesis. Figure 2c and d show high-resolution (HR)STEM bright-field (BF) images of a pristine nanotube in atomic resolution and a cross-section lamella prepared via focused ion beam (FIB) from one such nanotube. The lattice model of the MLC compound SmS-TaS_2_ is overlaid on the HRSTEM image. Careful analysis of the atomic arrangement reveals tantalum and sulfur atoms coordinated in trigonal prismatic fashion in the TaS_2_ layer and the SmS 100 plane in a distorted rocksalt configuration. The cross-section image in Figure 2d reveals the SmS 110 direction and the S atoms in the rock salt lattice. Figure 2e shows the atomically resolved chemical mapping obtained with STEM-EDS of the nanotube cross-section, revealing the alternating layers of samarium and tantalum.

More recently, the focus turned to MLC nanotubes consisting of four elements, especially alloying the rocksalt lattice and the anion sites (S/Se).^43^ One remarkable property common to different MLC nanotubes is the hyperperiodicity of (*O-T*)(*O-T*)′ layers, whereby the (*O-T*)′ layer (armchair configuration) is titled 30° with respect to the adjacent (*O-T*) layer (zigzag)—see Figures 2c and S2.^26,44,45^ This hyperperiodicity is believed to be a manifestation of yet another strain relaxation mechanism in such nanotubes.

## Stoichiometry of MLC Nanotubes vs Bulk Material

As stated above, the deviation from stoichiometry in MLC compounds is expressed through the term 1 + *y* = 2*a*_*T*_/*a*_*O*_ (0.08 < *y* < 0.32). Figure S3 shows a schematic representation of an MLC bulk (a) and a nanotubular structure (b). For the case of nanotubes with (*O-T*) periodicity, the stoichiometry is determined also via the respective diameter of each of the slabs (see schematics in Figure S3b). The TaS_2_ makes always the outer layer and as is clearly seen in Figure S3b, if the circumference of the SmS wall is taken as *L*_SmS_ = 2π*R*_SmS_ that of the TaS_2_ layer is – *L*_TaS2_ = 2π*R*_TaS2_ = 2π(*R*_SmS_ + *d*), where *d* is the spacing between the SmS and TaS_2_ layers and *R*_*O*__,*T*_ is the radius of the *O* and *T* layers. Representing this feature in terms of stoichiometry, one could conclude that 1 + *y*_*NT*_ in (SmS)_1+*y*_TaS_2_ nanotube is smaller than in bulk (SmS)_1.19_TaS_2_ crystals. Therefore, for nanotubes one can write 1 + *y*_*NT*_ = (2a_*T*_/a_*O*_)/(*L*_TaS2_/*L*_SmS_) = 1.19/(1 + *d*/*R*_SmS_). Taking *d* ∼ 0.6 nm and *R*_SmS_ = 50 nm, 1 + *y*_*NT*_ = 1.176 instead of 1.19, i.e., 1.1% deviation from the overall bulk MLC stoichiometry. For an inner layer of the nanotube with *R*_SmS_ = 10 nm, the deviation from stoichiometry is 1 + *y*_*NT*_ = 1.122, which represents a significant deviation (11%) from the bulk material. This deviation also means that the overall excess charge of the *O* layer in the nanotube is reduced with appreciably smaller charge transfer. Therefore, the Ta 5*d*_*z*^2^_ level near the Fermi level is hole-rich (electron deficient) in the nanotube compared to the bulk MLC endowing the nanotube higher electrical conductivity than the bulk material. The actual deviation from stoichiometry (1 + *y*_*NT*_) can be smaller since it can be partially compensated by defects, antisites, atom transfer from one layer to the other.

## Stability of Nanotubes, General

The chemical selectivity and stability of MLC nanotubes versus their macroscopic flakes and also with respect to the binary compounds of their constituent atoms at low and mostly at elevated temperatures are discussed next. Another issue, which is not disconnected from the previous one, is the charge transfer from the MX slab to the TX_2_ slab.

The chemical reactivity and high-temperature stability of fullerene-like (IF) nanoparticles of WS_2_, MoS_2_, and NbS_2_ was investigated in inert and oxygenated atmospheres in comparison with the bulk crystallites.^46^ It was found in an earlier study that the decomposition temperature of IF-WS_2_ nanoparticles (containing also 10% nanotubes) was about 50 °C lower than that of the bulk material in inert atmosphere, i.e., 1250 vs 1300 °C for WS_2_. However, a direct comparison with density functional theory (DFT) calculations of the high temperature behavior of inorganic nanotubes has not been done so far. Note, however, that phonon contributions to thermodynamic properties of different nanotubes as a function of temperature was estimated using zone folding approximation.^47^

## Chemical Selectivity of Rare-Earth Based MLC Nanotubes

It is well-known in the literature that the distribution of sulfur and selenium in the TMDC lattice is random at elevated temperatures.^48,49^ So far little has been done to elucidate the phase behavior of MLC at elevated temperatures,^50^ let alone nanotubes thereof. While much effort has been invested over the last 30 years to perfect the synthetic routes of different (metastable) nanostructures, the question of their stability limits and decomposition pathways remains largely obscure. One major obstacle in this respect is the difficulty of comparing the experimental data to the *ab initio* calculations. Owing to the large unit cell of the “approximant” and the heavy atoms, *ab initio* calculations of MLC nanotubes are currently beyond reach. This also means that the simpler force-field analysis is of limited use under these circumstances because it cannot be parametrized against DFT calculations. Furthermore, *ab initio* calculations are generally limited to zero Kelvin (see, however, ref (51)), and they are unable to address the high-temperature stability of nanotubes, accurately.

**Figure 3:**
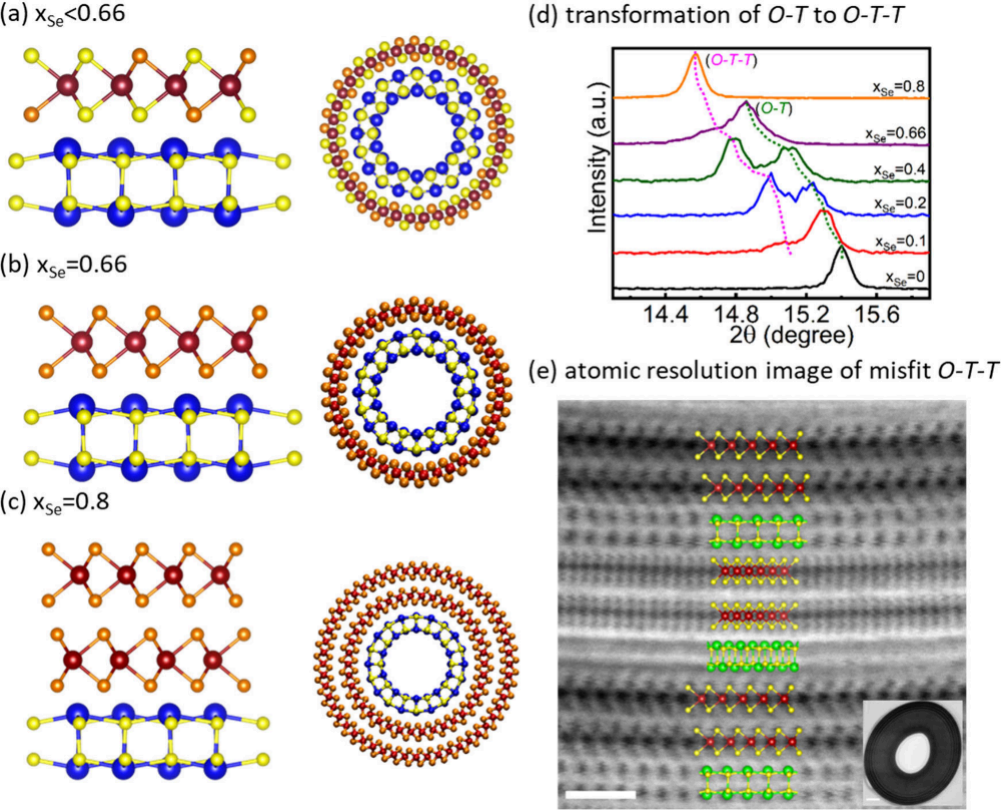
(a) Schematic rendering of the high temperature chemical affinity of MLC (*O-T)* layered structure with La exclusively binding to S (yellow) in the lattice and the corresponding nanotube geometry. (b) For *x*_Se_ = 0.66, notice the exclusive binding of Se (orange) with Ta and La with S. (c) Structure of MLC with (*O-T-T*) arrangement at the composition *x*_Se_ = 0.8 with selective binding Se with Ta and La with S. (d) Powder XRD pattern of LaS-TaS_2_ with varying *x*_Se_ showing the gradual conversion of MLC (*O-T*) type lattice into (*O-T-T*) type lattice. (e) Atomic resolution HAADF-STEM image of nanotube cross-section lamella of the composition *x*_Se_ = 0.8 showing the (*O-T-T*) structure, a schematic model has been overlaid, scale bar 2 nm (Adapted from ref (2) with modifications, copyright 2024 Authors).

The stability and chemical selectivity of nanotubes belonging to the alloyed MLC La(S,Se)-(Ta(S,Se)_2_)_*n*_ was recently elucidated.^2^ Surprisingly, the reaction of La, Ta, S and Se (up to 1100 °C) yielded MLC nanotubes with lanthanum atoms bonded exclusively to sulfur atoms and the selenium to tantalum atoms (as shown in Figure 3). Consequently, for *x*_Se_< 0.66, where *x*_Se_ + *x*_s_ = 1, the chemical composition of the nanotubes (and flakes) could be presented as LaS-TaSe_2*x*_S_2(1–*x*)_. At *x*_*Se*_ = 0.66 the amount of sulfur was sufficient to form nanotubes with the (*O-T*) formula LaS-TaSe_2_. Upon increasing the *x*-value even further, partial starvation with respect to sulfur led to the formation of LaS-TaSe_2_-TaSe_2_ (*O-T-T*) nanotubes (and flakes). Figure 3b–c presents a schematic rendering of the nanotubes with (*O-T*) structure LaS-TaSe_2*x*_S_2(1–*x*)_ for *x* < 0.66; LaS-TaSe_2_ for 0.66 < *x* < 0.8 and LaS-(TaSe_2_)_2_ (*O-T-T*) for *x* = 0.8 and little beyond. In fact, the transitions are not abrupt, as can be visualized by the X-ray diffraction (XRD) patterns displayed in Figure 3d. Here the (*O-T-T*) peak is visible already for *x* = 0.2, but the (*O-T*) peak is diminished entirely for *x* = 0.8, indicating smooth transition from (*O-T*) to (*O-T-T*) structure due to the gradual starvation with respect to sulfur. Figure 3e shows a high-resolution STEM (HRSTEM) image of a lamella cut from an (*O-T-T*) nanotube prepared from sample with x = 0.8. Not only the (*O-T-T*) structure is confirmed, but also the hyperperiodicity, i.e., LaS-(TaSe_2_)_2_, LaS-(TaSe_2_)_2_′ structure with 30° tilt between the two adjacent (*O-T-T*) pairs is validated.

Ultimately, when the sulfur content was limited further, the MLC nanotubes (and flakes) became unstable and no MLC nanotubes (or flakes) based on pure selenium, i.e., LaSe-TaSe_2_ could be found. To understand this surprising chemical selectivity, one should bear in mind that the stability of MLC is enabled by the charge transfer from the LaS to TaSe_2_.^52^ Once an MLC superstructure (*O-T-T-T*) is formed, the middle TaSe_2_ does not contact any LaS layer, preventing efficient charge transfer and leading to a reduced stability of the superstructure. In this case, the MLC was found to decompose into TaSe_2_, pure selenium, and some binary lanthanum chalcogenide compound. Here too, the hyperperiodicity of the form (*O-T*)(*O-T*)′ and (*O-T-T*)(*O-T-T*)′ with 30° inclination of the (*O-T*)′ layer with respect to the adjacent (*O-T*) one was common in the nanotubes. The conspicuous chemical selectivity of the lanthanum atom toward sulfur and the selenium atom toward tantalum at elevated temperatures was recently found to go beyond lanthanum and encompasses other rare earth atoms.

In the present perspective, the stabilities of (LaS)_1.14_TaS_2_ and (SmS)_1.19_TaS_2_ nanotubes are studied as a function of the synthesis time and temperatures. A preliminary study on this topic was recently published,^1^ and is largely expanded by including synchrotron-based X-ray absorption (XAS) and diffraction anomalous fine structure (DAFS) techniques. This expansion allows for a more profound understanding of the decomposition process of the MLC nanotubes at elevated temperatures.

## The Thermal Stability of MLC Nanotubes at Elevated Temperatures Studied by Synchrotron-Based X-ray Techniques

Recently, the thermal stability of SmS-TaS_2_ and LaS-TaS_2_ nanotubes were investigated.^1^ In the first series of experiments (Table S1), the nanotubes were synthesized at different temperatures (from 800 to 975 °C). In the second series of experiments, the materials were synthesized at the optimal temperature (825 and 875 °C for the Sm- and La-based nanotubes, respectively) for different time-intervals. In the third kind of experiment, the MLC nanotubes were prepared in quartz ampules at their optimal condition, i.e., 825 and 875 °C for 4 h for the Sm- and La-based MLC nanotubes (Figures 2a and S1, respectively. The as-synthesized nanotubes went through an annealing process above 1000 °C (see Table S1 for the description). The laboratory based XRD patterns for both temperature and time series of SmS-TaS_2_^1^ and LaS-TaS_2_ (Figures S4 and S5) MLCs show highly preferred (00*l*) orientation and also indicate the gradual structural transformation from (*O-T*) to (*O-T-T*) and then to (*O-T-T-T*). The preferred orientation overshadows the (*0kl*) reflections, making it difficult to analyze the conversion of nanotubes into flakes.

Diffraction patterns of the nanotubes mainly consist of the sharp 00*l* reflections that are associated with the interlayer spacing and the *hk*0 reflections that are heavily streaked due to translation stacking disorder associated with the direction perpendicular to nanotube axis (Figure S2).^53−60^ Due to the lack of the out-of-plane symmetry of the curved nanostructures and circumferential disorder between layers, the diffraction patterns of the nanotubes do not exhibit the distinct and sharp *hkl* reflections (with *l* ≠ 0, namely *h0l* and *0kl*) that distinguish them from the bulk particles—see Table 1. These diffraction features first observed in carbon nanotubes using electron microscopy were also observed in MLC nanotubes with the help of synchrotron-based subμm XRD.^1^ Spatially dispersing the SmS-TaS_2_ nanotubes and SmS-TaS_2_ flakes on the SiN grid away from each other, it was possible to get the XRD patterns from single MLC nanotubes and flakes, thus revealing their differences—see Figure S6. Generally, while the 00*l* peaks belong to both the nanotubes and flakes, the 0*kl* peaks can be assigned solely to the flakes. These results provide the tools for analyzing the temperature and time dependence of the nanotubes, summarized in the next series of experiments described below.

**Table 1:** Reflection Conditions for the Most Common Phases in the Powder Containing Bulk, Single-Layer 2D Materials, and Their Nanotubes^b^

Reflections	Bulk particles/Multilayered flakes (platelets)	Nanotubes	Single layers
00*l*	+	+	–
*hk*0	+	+	+
*h*0*l*	+	–a	–
0*kl*	+	–a	–

a This peak is not absolutely forbidden for large diameter tubes, where the curvature is small.

b The *h*0*l* and 0*kl* reflections will be absent for nanotubes.

Adopted from reference (1), copyright 2024, CC-BY 4.0, Authors.

In the following experiments, the products were analyzed via electron microscopy, X-ray powder diffraction, synchrotron-based X-ray diffraction, X-ray absorption spectroscopy (XAS), and diffraction anomalous fine-structure (DAFS).

The synchrotron-based XRD analysis of SmS-TaS_2_ and LaS-TaS_2_ as a function of the synthesis temperature is discussed in the SI and shown in Figures S7 and S8, respectively. This analysis shows clearly that the abundance of the nanotubes goes down with temperature. It also shows a gradual transformation from the (*O-T*) superstructure to the (*O-T-T*) superstructure, with the La-based MLC being more prone to this transformation. Above 1100 °C a third process, i.e., decomposition of the MLC phases into binary sulfides and partial oxidation are observed. These transformations are also discussed further below.

## Effect of the Synthesis Temperature Studied via X-ray Absorption Spectroscopy (XAS) and Diffraction Anomalous Fine-Structure (DAFS) Measurements

**Figure 4:**
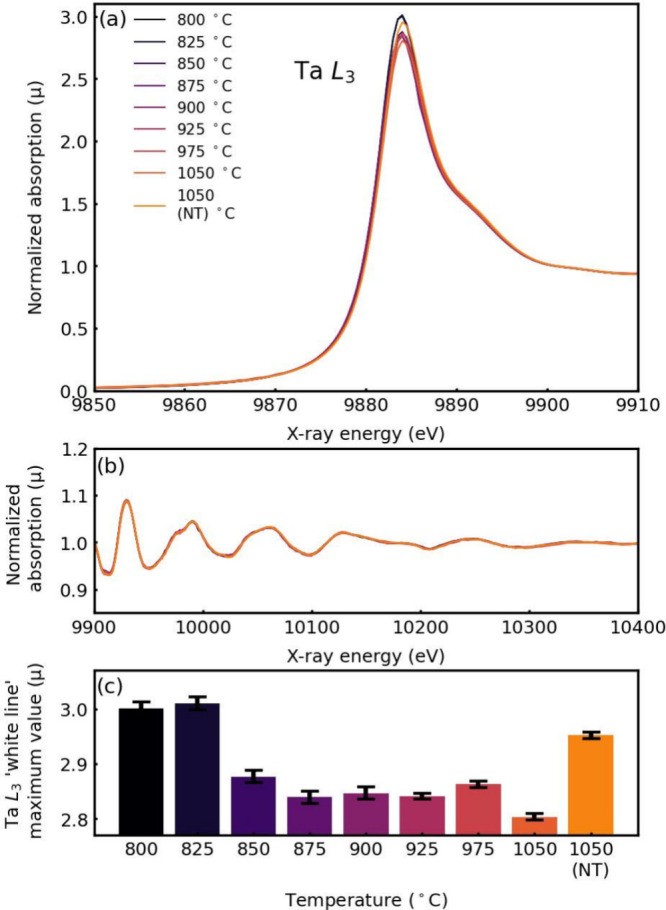
X-ray absorption spectra in the vicinity of the Ta L_3_ edge of SmS-TaS_2_ samples achieved at different temperatures: XANES (a) and EXAFS (b) regions. The (c) shows the height of the Ta L_3_ white line as a function of temperature.

XAS spectra measured in the transmission mode of the SmS-TaS_2_ samples (absorption) for the temperature series in the vicinity of the Ta L_3_ edge are presented in Figure 4. From the extended X-ray absorption fine structure (EXAFS) region (Figure 4b) it is seen that there is no significant change in the oscillation amplitudes with the temperature of the synthesis, indicating that the local structure of the tantalum atom (bond distance, etc.) remains almost the same. The white line of the XAS spectrum at X-ray absorption near edge (XANES) region is due to electron excitation from the occupied 2*p* core level of tantalum into its empty 5*d*_*z*_^*2*^ state near the Fermi level. It is noticed that the Ta L_3_ white line intensity decreases with increasing temperature (Figure 4c). One can see a strong step change at 850 °C, and then a plateau at the temperature range 850–975 °C. Then at 1050 °C the intensity falls again. The drop in the intensity of the white line can be attributed to the filling of the Ta 5*d*_*z*_^*2*^ state with electrons, which is indicative of the nanotubes-to-flakes transformation with temperature, as well as (*O-T*) to (*O-T-T*) transformation and decomposition of the MLC into binary TaS_2_. These transformations are discussed in connection with the XRD analysis (Figures S7 and S8) in the SI text. The block 1050 (NT) summarizes the results of an experiment in which the product was prepared at 825 °C (>50% nanotubes abundancy) and subsequently annealed at 1050 °C for 4 h. Remarkably, the high intensity of the white line after 1050 °C annealing indicates that the decomposition of the nanotubes is retarded, compared to the direct synthesis of the product at temperatures above 850 °C. One plausible explanation is that the chlorine gas, which drives the CVT reaction and consequently also the phase transformation, has been frozen-out during the intermediate cooling of the ampule to room temperature between the synthesis (at 825 °C) and the annealing 1050 °C. Another plausible explanation is that the reaction product has decomposed into binary TaS_2_ and other byproducts, like Sm_2_Ta_3_S_2_O_8_ upon annealing.^48^ Being richer in free carriers (holes), pure TaS_2_ would lead to a stronger white line compared with the MLC. This point is discussed further below.

However, it is necessary to analyze the DAFS data in order to clean the nanotube XAS signal from the influence of other phases present in the samples. The DAFS technique is based on measurements of the diffraction peak intensity in the vicinity of the X-ray absorption edge.^61−65^ Contrary to conventional XAS,^66,67^ DAFS allows measuring the XAS-like signal from certain phase or crystallographic site separately by choosing the proper diffraction peak, thus providing phase- and site-selectivity. Thus, by choosing the diffraction peak from a certain phase within the powder mixture, one will get spectroscopic information solely from this phase from DAFS measurement. In the present DAFS analysis logarithmic dispersion relation (LDR) approach proposed by Kawaguchi^64,65^ was used. Detailed description of the DAFS experimental setup (Figure S9), as well as the procedure for data analysis can be found in the Supporting Information.

**Figure 5:**
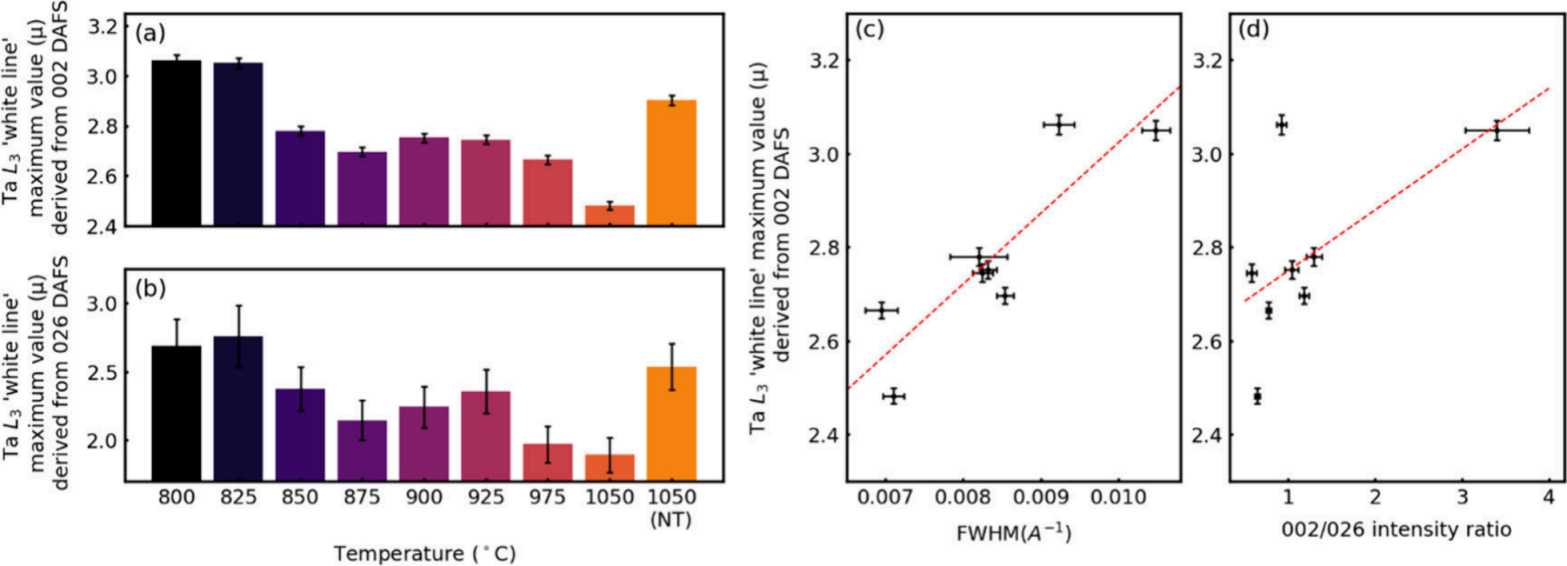
White line intensities derived from the 002 (a) and 026 (b) DAFS reflections of SmS-TaS_2_ as a function of temperature; (c) and (d) represents the white line intensity of the 002 reflection on the fwhm and nanotube abundance (002/026), correspondingly.

According to the discussion above (and Table 1), one can use the 026 reflection for getting the spectroscopic information from the MLC flakes and the 002 reflection for receiving the information from both tubes and platelets with the DAFS method. An example of the DAFS experimental data for SmS-TaS_2_ is presented in Figure S10, where the variation of the diffraction intensity of these reflections in the vicinity of the Ta L_3_ edge is clearly visible. Figure S11 presents the DAFS (reflection) spectrum of the 002 and 026 diffraction peaks intensity of SmS-TaS_2_ samples at different temperatures, and Figure 5 represents its analysis. Figure 5a and b shows block diagrams of the white line (DAFS) intensity derived from the 002 and 026 reflections as a function of temperature, correspondingly. The intensity of the 026 reflection is appreciably weaker in comparison to the 002 peak and also overlaps partially with the 0010 peak (Figure 5b). Therefore, the measured intensity of this reflection has a large error bar. Here too, the white line is due to electron excitation of the occupied 2*p* core level of tantalum into its empty 5*d*_*z*_^*2*^ state near the Fermi level. The reduced intensity of the 002 reflection white line of SmS-TaS_2_ upon increasing the temperature (Figure 5a) suggests that the Ta 5*d*_*z*_^*2*^ level is being filled with electrons and the free carrier (holes) density goes down with increasing growth temperature. This finding is compatible with the transformation of the substoichiometric nanotubes into MLC flakes, which incorporates extra electron-rich samarium (in the 4*f*^*6*^ level). It is also compatible with the transformation of the (*O-T*) phase into (*O-T-T*), where the charge transferred from the SmS (*O*) is shared between two TaS_2_ (*T*) layers, as seen in the 1050-nanotube sample. Remarkably, the intensities of the “white” line of the sample annealed at 1050 °C after being synthesized at 825 °C (1050 (NT)) are high indicating that the nanotubes are not easily transformed into flakes in this process. Alternatively (or conjointly), it may indicate that the nanotubes have been decomposed into TaS_2_ and other side-products.

However, as shown in the lower panel of Figure 5b, the intensity of the white line associated with the 026, which belongs exclusively to the MLC flakes, goes down as well with the temperature. To explain this phenomenon, one must evoke another mechanism which could contribute to the white line intensity i.e., the surface density of the tantalum atoms. One such explanation for the weakening of the white line of the 026 DAFS peak with temperature is related to the effect of the surface atoms. It has been established in the past^68^ that surface effects play an important part in the white line intensity for nanoparticles. The intensity of the white line of the Pt surface atoms was larger in comparison to atoms in the bulk. Therefore, the white line from both MLC flakes and nanotubes should, in principle, depend on the particle size because the influence of the surface atoms becomes significant at small particle sizes. In addition, notwithstanding the large error bars, Figure 5c and d demonstrate that the white line intensity (straight line is a guide to the eye) increases with the abundancy of the nanotubes as manifested via the FWHM (Figure 5c) and the 002/026 intensity ratio. Therefore, the transformation of the nanotubes into large flakes at higher temperatures leads to a reduced white line intensity, which is ascribed, among others, to a smaller surface density of tantalum atoms in the flakes compared with the nanotubes. Again, the increased intensity of the white line of the 1050 (NT) can be putatively ascribed to transformation of the MLC into binary TaS_2_ and other byproducts. Here, the explanation of the white line intensity behavior is of qualitative character and partially based on XRD observations; quantitative considerations require XAS computer simulations based on nanotube structural models, which are beyond the focus of this perspective.

## Effect of the Synthesis Time

Figure S12 in SI summarizes the results of the annealing time (1 to 90 h) of the LaS-TaS_2_ MLC with synchrotron-based XRD. First, the obvious nanotubes-to-flakes transformation with annealing time is clearly observed. Most outstandingly, however, a clear transformation of the (*O-T*) structure into (*O-T-T*) after 8 h synthesis and subsequent transformation of the (*O-T-T*) into (*O-T-T-T*) phase after 16 h synthesis time is omnipresent. This autocatalytic transformation is not exclusive to this system and is common in intercalation chemistry including Li-ion batteries.^69,70^

**Figure 6:**
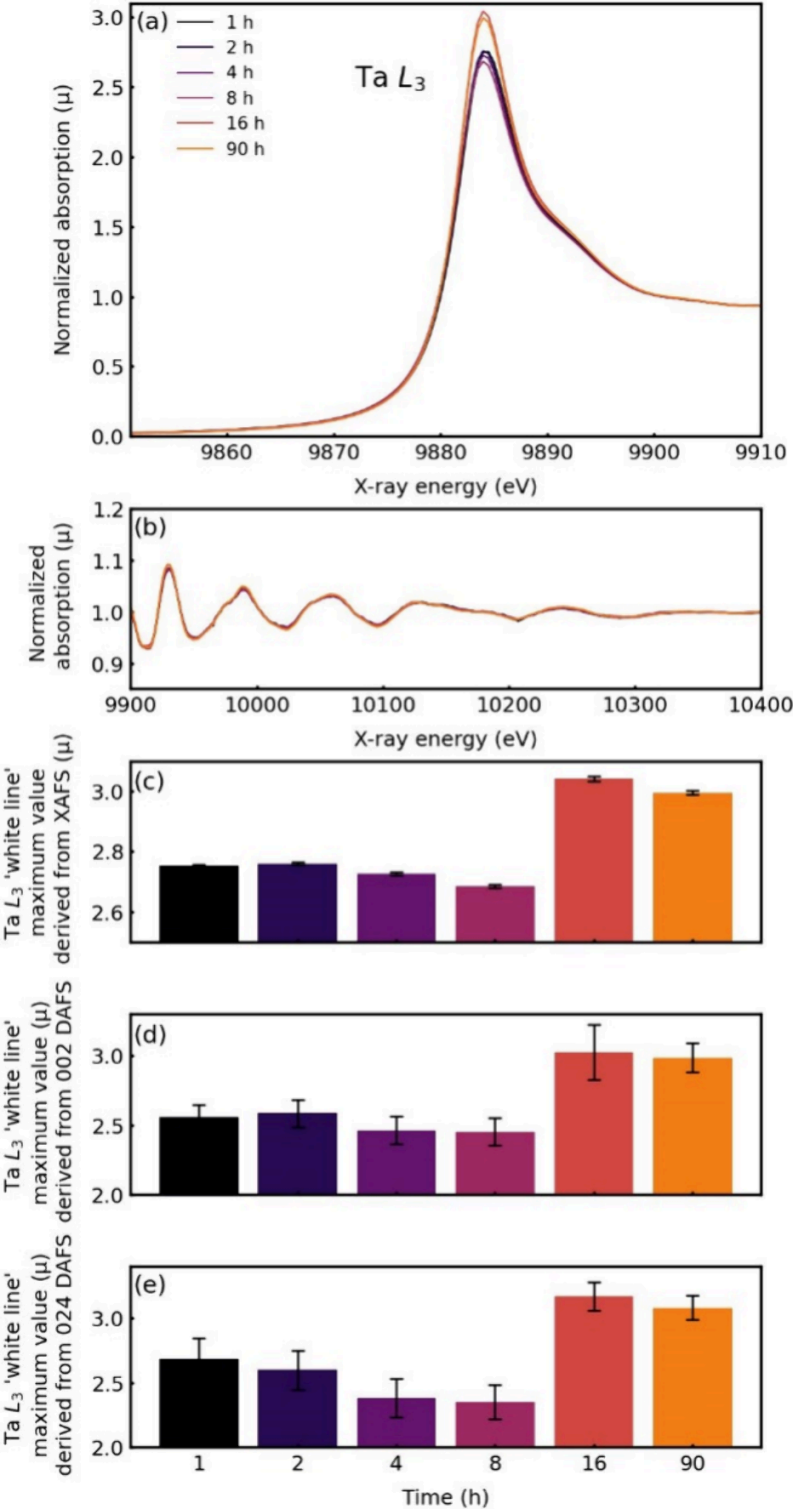
X-ray absorption spectra in the vicinity of the Ta L_3_ edge of LaS-TaS_2_ samples obtained at 875 °C with varying reaction time: XANES (a) and EXAFS (b) regions. (c) shows the height of the Ta L_3_ white line as a function of reaction time; white line intensities of the DAFS spectra derived from the 002 (d) and 024 (e) peaks as a function of time.

Figure 6 presents the X-ray absorption near the Ta L_3_ edge for LaS-TaS_2_ as a function of the reaction time. In analogy to the case of SmS-TaS_2_, the EXAFS region (Figure 6b) shows no significant change of the oscillation amplitudes with heating time, indicating that the local structure (bond distance, type, and number of neighbors) remains almost unchanged with annealing time. The block diagram in Figure 6c shows that the intensity of the white line increases slightly after 2 h and then gradually decreases up to 8 h, which suggests that the charge transfer from the LaS slab to the TaS_2_ slab goes down with annealing time, for reasons explained above for the temperature series of the SmS-TaS_2_ MLC (transformation of the MLC tubes into flakes). However, a sharp rise in the intensity of the white line occurs after 16 h followed by a small decline in its intensity after 90 h annealing time. This significant intensity variation of the white line is indicative of a major transformation in the La-based MLC after 16 h. In particular, this intensity rise is attributed to a significant deintercalation of the LaS layer from the (*O-T*) lattice periodicity to form a new (*O-T-T*) and sequentially to (*O-T-T-T*) periodicity as seen on the XRD data (Figures S5 and S12b and c). The transformation of (*O-T-T*) to (*O-T-T-T*) periodicity is further confirmed by HAADF-STEM imaging (Figure S13). Furthermore, the high temperature transformation of the MLC phase into binary TaS_2_ and LaS_*x*_ and other byproducts could also make an important contribution to this variation in intensity variation.

All these processes imply that the LaS → TaS_2_ charge transfer is greatly reduced and hence the free hole concentration of the 5*d*_*z*^2^_ level of Ta increases sharply with the synthesis time. The same trend is observed in the block diagram of Figure 6d and 6e, which presents the strength of the white lines obtained from 002 and 024 DAFS, correspondingly (see also Figure S14). The similarity between the independent XAS data (Figure 6c) and the DAFS data (Figure 6d and e) is not accidental, offering a similar interpretation.

## Conclusions and Outlook

Like most other nanophases, nanotubes formed from inorganic compounds based on a layer structure are metastable and are converted to macroscopic crystallites upon prolonged heating. This view is validated also in the case of nanotubes from misfit layer compounds (MLC), which are the topic of the present perspective. Using electron microscopy and mostly synchrotron-based X-ray reflection and absorption techniques (XRD, XAS, and DAFS), the mechanisms of such transformations were investigated in hitherto unknown detail. First, the chemical selectivity of the constituent metal atoms of the MLC nanotubes toward the chalcogen atom is examined. Notwithstanding the high temperature of the synthesis (up to 1000 °C), sulfur atoms are shown to bind exclusively to the rare-earth atom of the rocksalt slab, while selenium binds solely to the tantalum of the hexagonal TX_2_ slab. This surprising selectivity is attributed to the large enthalpy of formation of the MLC nanotubes. Using selection rules for the XRD and diffraction anomalous fine-structure (DAFS) reflections of nanotubes as well as X-ray absorption (XAS), the common transformation of sulfide-based rear earth MLC nanotubes into microscopic flakes is investigated. The conspicuous transformation of (*O-T*) nanotubes (and flakes) into (*O-T-T*) and sequentially (*O-T-T-T*) phases at elevated temperatures is investigated. This autocatalytic reaction is in fact a reminiscent of the electrocatalytic deintercalation of lithium from the anode material in Li-ion batteries. Furthermore, partial oxidation of the product at elevated temperature was observed and discussed. Annealing at higher temperatures (>1100 °C) and for longer periods of time (up to 90 h) leads eventually to the decomposition of the ternary MLC into binary metal-sulfide phases.

While no immediate application for MLC nanotubes is visible in the near future, they may offer intriguing observations and potential exploitation in the future as anode materials for intercalation batteries,^71^ electrocatalysis, for electronics,^72^ thermoelectrics,^73^ and in quantum technologies.^74^ Their catalytic and electrocatalytic properties are yet another aspect to be explored. Their limited stability under an oxidative environment limits their uses under cathodic conditions, like hydrogen evolution reaction, or for CO_2_ sequestration.

## Supporting Information Available

Sample description for LaS-TaS_2_ (Table S1), electron microscopy images, XRD of time and temperature series LaS-TaS_2_ series, synchrotron experiments description of single nanotube diffraction, and description and analysis of XAS and DAFS spectral features (PDF)
