# Supplementary material for: Misfit Layered Compounds: Insights into Chemical, Kinetic, and Thermodynamic Stability of Nanophases
Source: Acc Chem Res. 2024 Nov 4;57(22):3243–53. doi: 10.1021/acs.accounts.4c00412 (PMC11580169; doi:10.1021/acs.accounts.4c00412)
Supplement: Supplementary file 1 — ar4c00412_si_001.pdf [file ar4c00412_si_001.pdf]

Supporting Information for

Misfit Layered Compounds: Insights into Chemical, Kinetic and Thermodynamic Stability of Nanophases

Azat Khadiev,[1#] M.B. Sreedhara,[2*#] Simon Hettler,[3,4] Dmitri Novikov,[1] Raul Arenal,[3,4,5] and Reshef Tenne[6*]

[1]Deutsches Elektronen-Synchrotron DESY, Notkestr. 85, 22607 Hamburg, Germany

[2]Solid State and Structural Chemistry Unit, Indian Institute of Science, Bengaluru, 560012 India

[3]Instituto de Nanociencia y Materiales de Aragon (INMA), CSIC-Universidad de Zaragoza, 50018 Zaragoza, Spain

[4]Laboratorio de Microscopias Avanzadas (LMA), Universidad de Zaragoza, 50018 Zaragoza, Spain

[5]ARAID Foundation, 50018 Zaragoza, Spain

[6]Department of Molecular Chemistry and Materials Science, Weizmann Institute of Science, Rehovot 7610001, Israel

# contributed equally

*corresponding authors: sreedhara@iisc.ac.in, reshef.tenne@weizmann.ac.il

Table S1. Description of various reaction parameters, temperature, time and transport agent for LaS-TaS_2 MLCs nanotubes preparation and post-annealing. A detailed set of experiments for SmS-TaS_2 and other optimization experiments refer to ref. 1.

Number	Sample name.	Reaction Temperature (in °C)	Reaction time (in h)	Transport agent
	**Temperature dependence reactions**			
1	$(LaS)_{1.14}TaS_2$	800 °C	4h	Cl
2	$(LaS)_{1.14}TaS_2$	825 °C	4h	Cl
3	$(LaS)_{1.14}TaS_2$	850 °C	4h	Cl
4	$(LaS)_{1.14}TaS_2$	875 °C	4h	Cl
5	$(LaS)_{1.14}TaS_2$	900 °C	4h	Cl
6	$(LaS)_{1.14}TaS_2$	925 °C	4h	Cl
7	$(LaS)_{1.14}TaS_2$	975 °C	4h	Cl
	**Time dependent reactions**			
8	$(LaS)_{1.14}TaS_2$	875 °C	1h	Cl
9	$(LaS)_{1.14}TaS_2$	875 °C	2h	Cl
10	$(LaS)_{1.14}TaS_2$	875 °C	4h	Cl
11	$(LaS)_{1.14}TaS_2$	875 °C	8h	Cl
12	$(LaS)_{1.14}TaS_2$	875 °C	16h	Cl
12	$(LaS)_{1.14}TaS_2$	875 °C	90h	Cl
13	$(LaS)_{1.14}TaS_2$	875 °C (slow cooling)	90h	Cl
	**Annealed $(LaS)_{1.14}TaS_2$ MLC nanotubes prepared at 875 °C**			
14	$(LaS)_{1.14}TaS_2$	1050 °C	4h	Cl
15	$(LaS)_{1.14}TaS_2$	1200 °C	96h	-

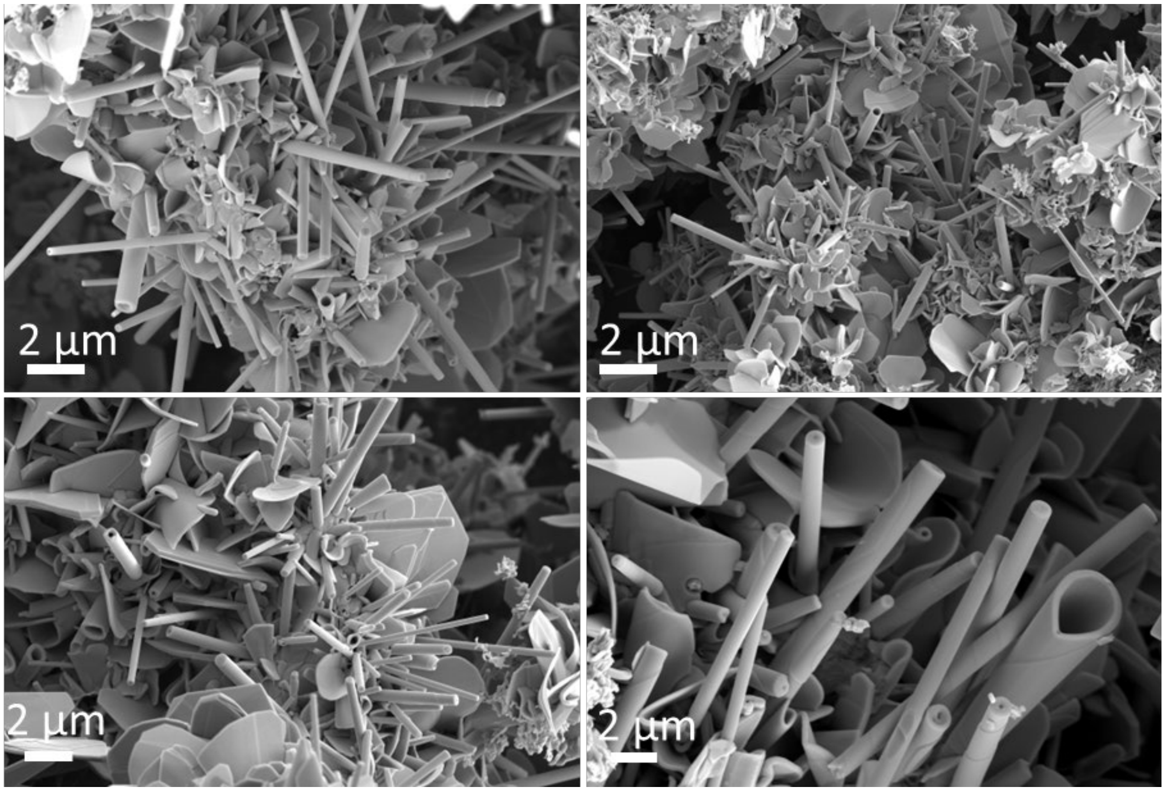

Figure S1. SEM image of LaS-TaS_2 nanotubes obtained by chemical vapor transport reaction at 875 °C, for 4 h using Cl as transport agent. The images are collected by distributing the solid powder on carbon tape. The nanotubes, nanoscrolls and nanoflakes constitute the entire powder.

<u>Electron diffraction analysis of MLC nanotubes</u>

Selected area electron diffraction (SAED) allows to investigate the crystal structure of materials. In contrast to TEM imaging, which typically gives information on a very local scale (few tens of nanometers), SAED permits to study an entire MLC nanotube. **Figure S2**b shows three SAED patterns and accompanying TEM images of MLC nanotubes that shed light on the relative orientation of the two different layered structures. During our studies, only few nanotubes could be found that exhibit a single orientation (or folding vector) for both subsystems throughout the entire nanotube, such as seen for the analysis of an YS-TaS_2 nanotube shown in **Figure S2** a-c. In the example SAED in Figure S2b, 4 pairs of spots are seen for the YS 110 reflections (marked by green circles) and 6 pairs of spots for the TaS_2 10.0 reflections (purple circles), which in direction of the NT axis agree with the YS 200 reflections (blue circles). This indicates that both subsystems crystallize in a single orientation, which is fixed with respect to the nanotube axis. The presence of this single orientation is confirmed by the STEM HAADF image in (c). Most of the nanotubes, however, show two sets of orientation with a 30º rotation between each other, which agrees with the found hyperperiodicity of (*O-T*)-(*O-T*)' by imaging discussed in the main article. As an example, Figure S2 c-e shows the analysis of a SmS-TaS_2 nanotube where two sets of reflections are found for both subsystems (exemplary marked for two SmS 110 reflections). Some nanotubes also

exhibit more than two folding vectors, such as the example SmS-TaS_2 nanotube shown in **Figure S2** g-i.

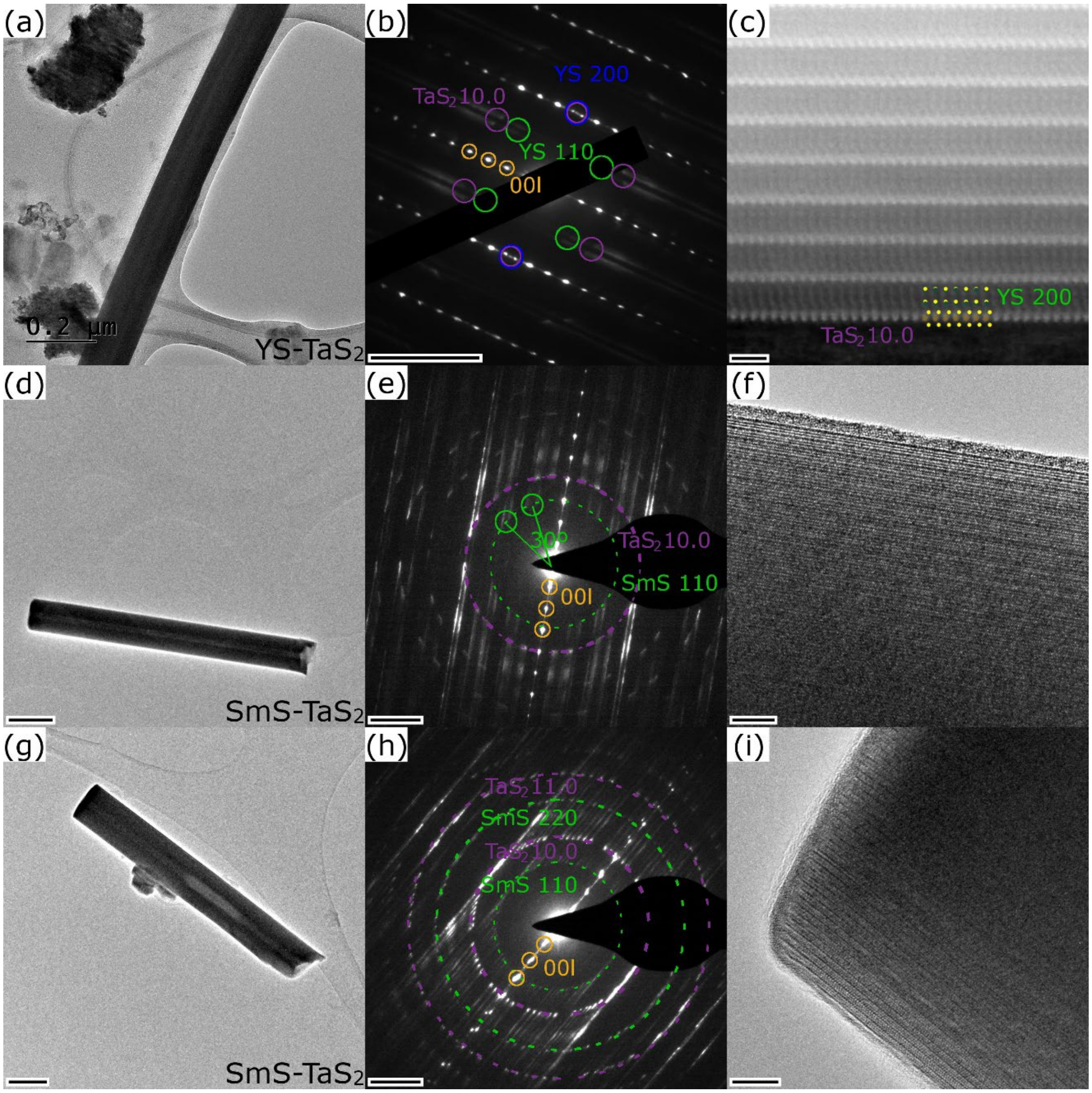

Figure S2. TEM and SAED analysis of three MLC nanotubes (a-c) This YS-TaS_2 nanotube shows a single folding vector, i.e. orientation of the two subsystems as indicated in the SAED pattern. Different reflections are marked in the image. The single orientation is also seen from the HAADF-STEM image in (c). (d-f) Most commonly, MLC nanotubes exhibit two folding vectors, which are rotated by 30º, causing a hyperperiodicity of the structure as exemplary demonstrated for this SmS-TaS_2 nanotube. (g-h) Some nanotubes, such as this one made of SmS-TaS_2 exhibit multiple folding vectors as seen from the numerous reflections for the two subsystems. Scale bars are (b) 5 nm^{-1}, (c) 1 nm, (d) 200 nm, (e,h) 2 nm^{-1}, (f) 5 nm (g) 100 nm, (i) 7 nm.

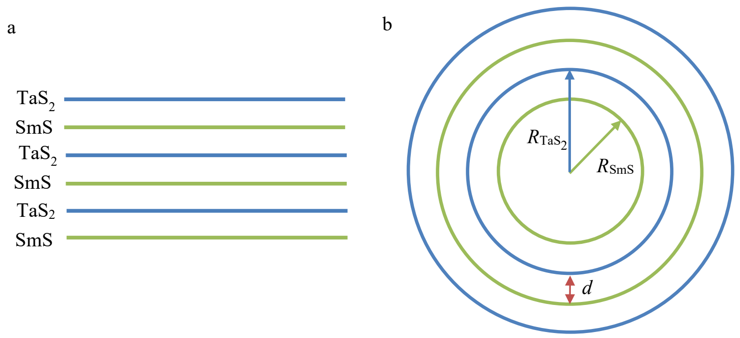

Figure S3. Schematic representation of bulk SmS-TaS_2 crystal (a) and nanotube (b). As revealed by experiments the outer layer is considered as TaS_2 and the inner layer is SmS. The crystallographic *b* direction coincides with the growth direction and the misfit *a* direction is along the circumference of the nanotube.

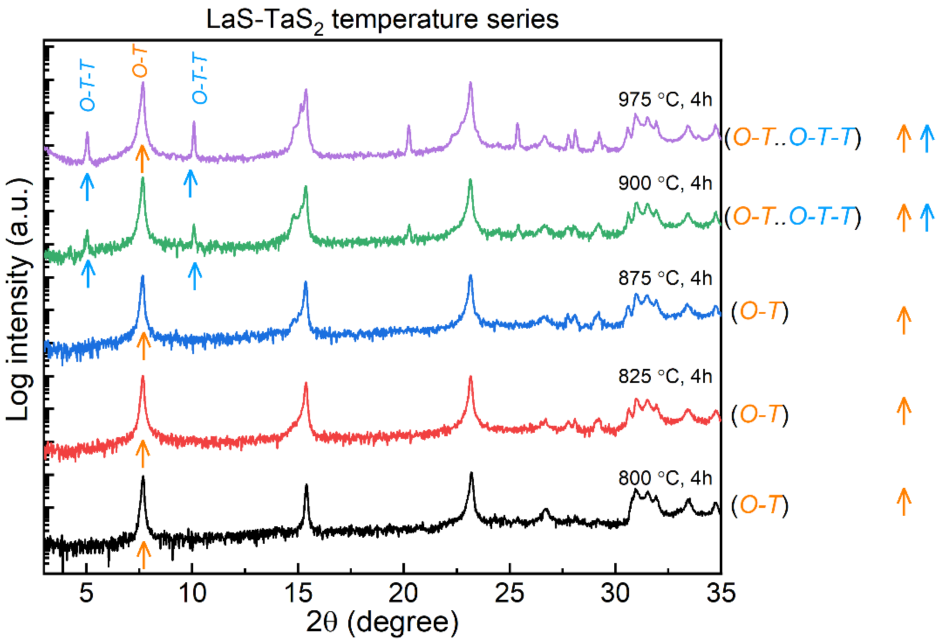

Figure S4. XRD patterns of LaS-TaS_2 temperature series: The pattern show highly preferred orientation along 00*l* with different periodicities corresponding to *O-T* (11.4 Å) and *O-T-T* (17.5 Å). The samples grown up to 875 ℃ show the reflection corresponding purely to *O-T* structure and above 875 ℃ show combination of *O-T* and *O-T-T* reflections.

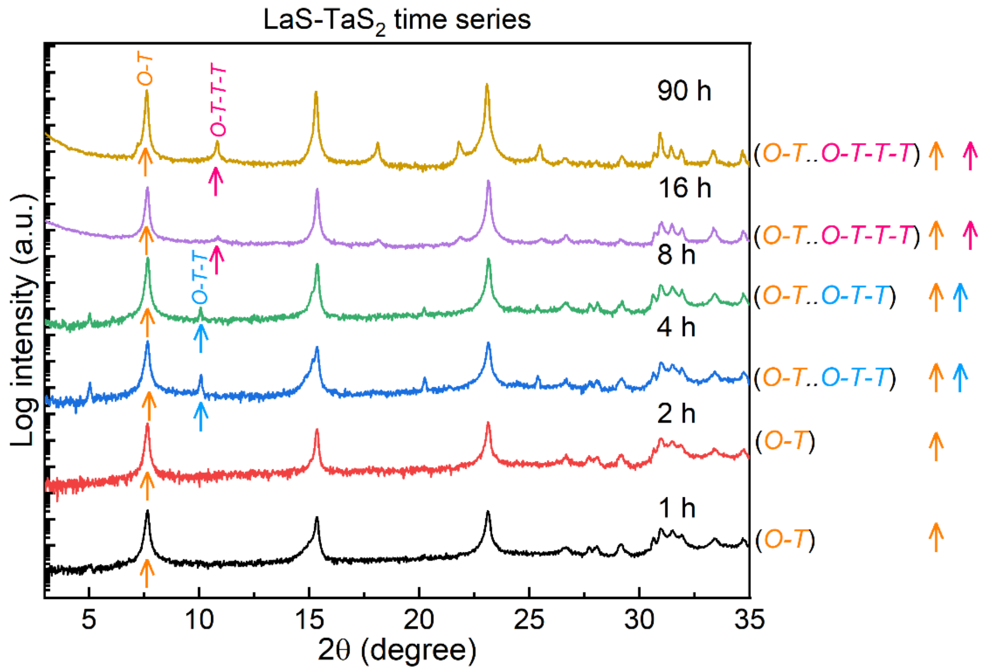

Figure S5. XRD patterns of LaS-TaS_2 time series: The pattern show highly preferred orientation along 00l with different periodicities corresponding to *O-T* (11.4 Å) *O-T-T* (17.5 Å) and *O-T-T-T* (24.4 Å). The samples grown up 2 h show the reflection corresponding *O-T*, between 4-8 h show combination of *O-T* and *O-T-T* and above 8h the pattern corresponds to *O-T* and *O-T-T-T*. The orange, blue and pink arrow indicate the reflections from (002) of *O-T*, (004) of *O-T-T* and (006) of *O-T-T-T,* respectively.

Single nanotube X-ray diffraction (XRD) with Synchrotron radiation

The sub-µm X-ray beam and XRD (denoted sometime- PXRD) experiments were performed at DESY P23 "In-situ X-ray diffraction and imaging beamline". The synchrotron X-ray beam focused with Beryllium Compound Refractive lenses[2, 3] (CRL) down to 0.8 × 3 µm size was used to distinguish the diffraction features of nanotubes and platelets. The SmS-TaS_2 sample grown at 825 °C for 4h was drop cast on a SiN membrane window in such a way that nanotubes and platelets were spatially separated few tens of micrometer aparton the membrane. The membrane was kept in vacuum during measurements and scanned with the help of the linear stage with a step of 2 µm. The XRD patterns were collected at each step with a DECTRIS PILATUS 1 M Si 2D X-ray detector. This approach allowed us to filter the diffraction images that belong purely to the nanotubes from those that also contain signals from MLC platelets. The design of the XRD experiments is described in detail in ref.[1]

Figure S6a displays the XRD pattern of a single nanotube with streaky diffraction spots, whose "tails" are perpendicular to the nanotube axis shown by the pink arrow. The summation of diffraction patterns from many nanotubes achieved at different steps leads to the pattern shown in **Figure S6**b**.** This image now resembles a powder pattern with spotty Debye-Scherrer rings. The advantage of this diffraction pattern is that it has better statistics in comparison to that in **Figure S6**a and, due to the image filtering, represents only the signal from the nanotubes. **Figure S6**c represents integrated XRD pattern from the full area of the sample where the signal both from nanotubes and platelets is present. **Figure S6**d shows azimuthally integrated 2D patterns (**Figures S6**a, b, and c, correspondingly**)** imitating the powder diffractogram from the single nanotube, a pure nanotube powder, and the whole sample containing both nanotubes and platelets. From **Figure S6**d it is clearly seen that the nanotube shows only strong 00*l* reflections and those of the

highly asymmetric *hk*0 (namely 020) planes. The sharp 026 reflection (at q=2.78 A^{-1}) occurs only for data collected from the full area of the sample indicating that it belongs to the MLC platelets. Thus, like WS_2 or chrysotile nanotubes, MLC nanotubes have several unique diffraction features, that distinguish them from the conventional 3D crystals and single 2D layers, that are based on the same structural units/layers (WS_2 nanotubes *vs* tungstenite (2H-WS_2), carbon nanotubes *vs* graphite etc.), namely the absence of *h*0*l* and 0*kl* reflections[4-7]. These features are summarized in **Table 1** of the main text.

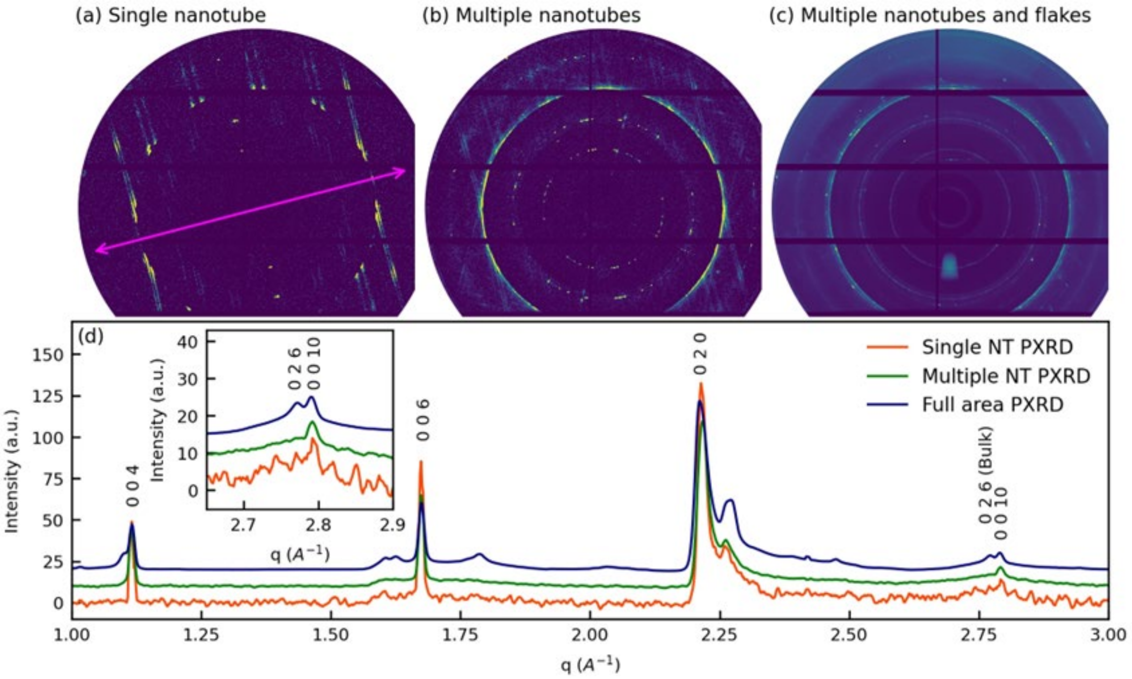

Figure S6. Comparison of sub-µm X-ray synchrotron patterns of a single nanotube; randomly oriented multiple nanotubes with that of bulk powder. (a) diffraction pattern of individual SmS-TaS_2 nanotube, the nanotube growth axis is marked with pink double headed arrow; (b) Combined diffraction pattern collected from many different nanotubes of the same composition in different orientations; (c) Integrated XRD pattern from the entire MLC powder sample containing both nanotubes and flakes. (d) Azimuthally integrated diffractograms from single nanotube, multiple nanotubes and powder sample containing nanotubes and flakes.[4]

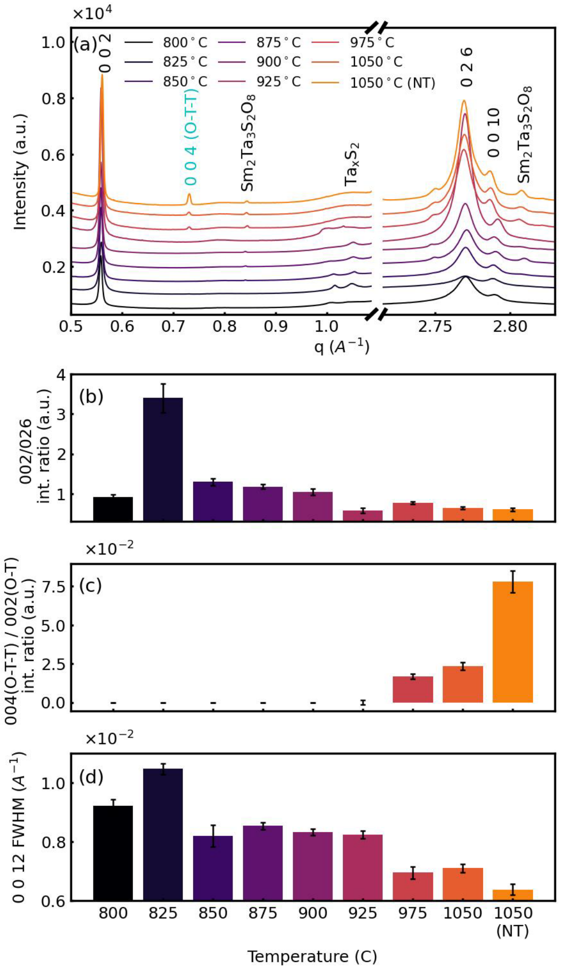

Figure S7. Enlarged areas of the synchrotron X-ray diffractograms of the as prepared SmS-TaS_2 samples and annealed samples (a), intensity ratios of 002/026 reflections (b), 004 (*O-T-T*) / 002 (*O-T*) peak intensity ratios (c) and full maximum half width (FMHW) of the 002 (*O-T*) diffraction peak (d).[1]

Figure S7 summarizes the results of the synchrotron-based XRD analysis of the SmS-TaS_2 product synthesized at different temperatures, while **Figure S8** summarizes the results for the LaS-TaS_2 product. **Figure S7**a presents the XRD patterns of SmS-TaS_2 prepared at different temperatures. The enlarged areas show the most prominent temperature-induced changes in the samples: the growth of the 026 diffraction peak and the appearance of the (*O-T-T*) structure. The 026 peak was found to be the most prominent peak for the SmS-TaS_2 MLC flakes. On the other hand, the 00*l* lines are common to the nanotubes and flakes and hence its intensity reflects the sum of the two morphologies. Therefore, the variation of the ratio between the 002/026 peaks was found to be most representative for the evolution of the nanotubes with annealing temperature and annealing time. **Figure S7**b shows a block diagram representing the evolution of this ratio with the temperature of the synthesis. Obviously from this diagram, the nanotubes become less abundant and transform into MLC flakes as the temperature of the synthesis rises. Simultaneously, another process takes place with increasing temperature of the synthesis, which is displayed in **Figure S7c**. As the temperature of the synthesis gets higher than 925 °C, the (*O-T*) phase is partially converted into (*O-T-T*) phase. Semi-quantitative phase analysis shows that the (*O-T-T*) mass fraction is below 6% of the (*O-T*) nanotubes and flakes even at the highest

temperature of 1100 °C. **Figure S7**d shows a block diagram representing the full width at half maximum (FWHM) of the 0012 peak as a function of temperature. It is clearly seen from this graph that the sample obtained at 825 °C has the largest 0012 (*O-T*) peak width, indicating that at this temperature the majority of the product consists of nanotubes having the smallest diameter (crystallite size) in comparison to the other samples. By analyzing the nanotube abundance data (**Figure S7**b) and nanotube diameter trend (**Figure S7**d) it is clearly seen that 825 °C is the optimal temperature for synthesizing the SmS-TaS_2 nanotubes providing the highest yield.

At synthesis temperatures above 1100 °C, a third process occurs i.e., the decomposition of the ternary MLC compound into binary sulfides i.e., TaS_2 and a binary samarium-sulfide phase. Another parasitic process, which occurs at elevated temperatures is the oxidation of the MLC tubes/flakes into oxides. This reaction is induced by the out-diffusion of residual water/oxygen remains from the ampoule walls, or simply by the high-temperature reaction of the MLC with the silica walls of the ampoule. Three phases were identified in the annealed samples: cubic(I) Sm_2O_3 (PDF 04-004-8958)/ orthorhombic $Sm_2Ta_3S_2O_8$ phases (PDF 04-011-1697) and $SmTaO_4$ (ICDD PDF-2 01-076-4605). The prime result of this study was that the nanotubes are gradually converted to MLC flakes at higher temperatures. Simultaneously, the (*O-T*) superstructure is converted into (*O-T-T*), and also slowly decomposes into binary compounds. To better understand these processes, a more complex analysis is required which is described below.

The diffractograms of the LaS-TaS_2 nanotubes at different temperatures show a similar behavior (**Figure S8**). The nanotube abundance goes down with temperature. The FWHM of the 0012 reflection (**Figure S8**d) shows that the optimal synthesis temperature is between 850-875 °C. However, in the case of the LaS-TaS_2 nanotubes, the 004 peak of the (*O-T-T*) phase starts to grow at lower temperature (900 °C) compared to the SmS-TaS_2 (975 °C). Most probably, this result indicates that LaS-TaS_2 is less stable to the *O-T-T* transformation in comparison to SmS-TaS_2.

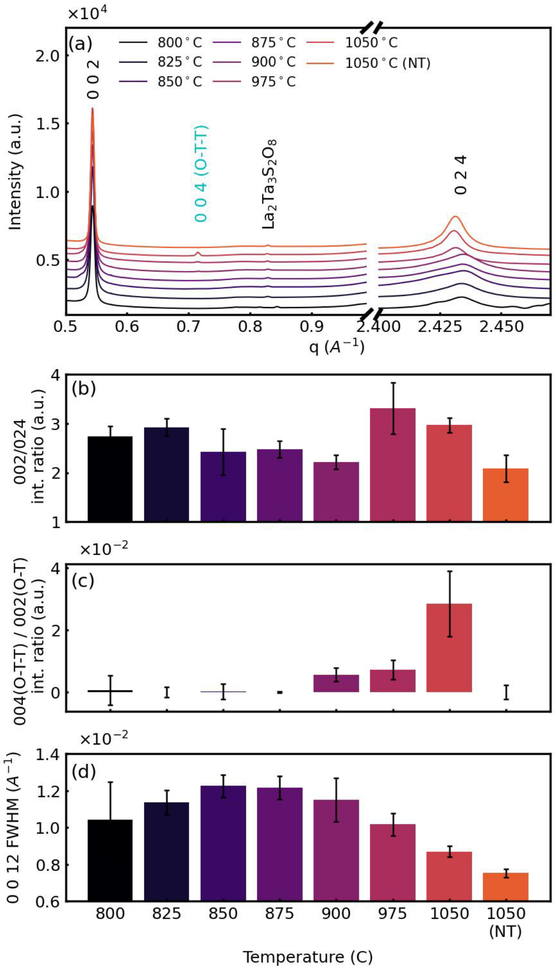

Figure S8. Enlarged areas of the diffractograms of the LaS-TaS_2 samples achieved and annealed at different temperatures (a), intensity ratios of 002/024 reflections (b), 004 (O-T-T) / 002 (O-T) peak intensity ratios (c) and 002 (O-T) diffraction peak FWHM (d).

Diffraction anomalous fine structure measurements (DAFS)

Experiment methods

MLC powders were mixed with cellulose binder (mixing ratio 1:3) and pressed with hydraulic press (2 ton) to produce 10 mm pellets suitable for analysis in transmission geometry. LaB_6 powder (NIST 660c) was also used as a standard for calibration of the detector distance. Mixing and handling of the powders were performed inside the Ar-filled glovebox (c(H_2O) and c(O2) < 1 ppm) to avoid possible oxidation of the samples. Before analysis, the sample pellets were placed between two Kapton foils (thickness: 50 microns) inside a special sample holder, forming a closed volume in order to avoid atmospheric contact during the measurements.

Experiments were performed at DESY P23 "In-situ X-ray diffraction and imaging beamline". The experimental set-up consists of entrance slits, a first intensity monitor, a rotating sample stage, a diffraction detector, and a second intensity monitor (**Figure S9**a and b) in the transmitted beam. Liquid N_2-cooled Double Crystal Monochromator (Si 111) was used for choosing the required

energy, and X-ray mirrors with Rh coating were used for harmonic rejection. Silicon avalanche photodiodes (APD) were used as intensity monitors for measuring the intensity of the incoming and transmitted X-ray beam scattered from the Kapton foils. The sample pellet was mounted on the OWIS DRTM 40 rotary stage and rotated with a speed of 180˚/sec during the analysis. An X-Spectrum Lambda detector with Si-based sensors (pixel size 55 microns, 1536x512 pixels) was used as a 2D diffraction detector. X-ray absorption (XAS) of the sample and diffraction were measured simultaneously at different X-ray energies in the vicinity of Ta L-III (9.881 KeV) X-ray absorption edge, thus producing more than 400 diffraction patterns (measurement points) near the X-ray absorption edge of each sample as shown in **Figure S10**. The diffraction pattern and X-ray absorption of the sample at a given energy were measured for a period of 10 sec. The energy of the incoming X-ray was also calibrated by measuring the absorption spectra of pure Ta (99,99 %, L-III edge) foil.

Analysis of the DAFS spectra

Diffraction Anomalous Fine Structure (DAFS) analysis was done by using a logarithmic dispersion relation (LDR) approach proposed by Kawaguchi[8, 9]. Before analysis, the diffraction data was corrected for polarization, Lorentz, and absorption factors (LPA). The linearity of the X-ray diffraction Lambda detector in the vicinity of the Ta L_3 edge was analyzed and corrected by measuring the LaB_6 standard powder, that didn't have absorption features in this X-ray energy range. The APD detector data was corrected for dead time[10, 11]. The linearity between the APD and Lambda detectors was also checked with LaB_6 powder at different attenuation ratios of the incoming X-rays; it was found that the APD/Lambda detectors' linearity was 0.9993 ± 0.0005. Integration of the 2D diffraction images and peak fitting was done by the pyFAI[12] and lmfit[13] software. XAS and DAFS-derived XAS-like spectra processing and analysis were done by the larch software[14]. An example of the experimental data collected for the SmS-TaS_2 sample synthesized at 825 ℃ is shown in Figure S6. During the energy scan, X-ray transmission and diffraction were simultaneously measured, thus providing the structural XRD and spectroscopic XAS information about the whole sample. Following the XRD data, LPA correction, and diffraction peak integration, one gets the DAFS spectra, which, in turn, after processing by the LDR approach, provide phase-selective XAS-like spectroscopic information from specific crystal or crystallographic site in the powdered sample.

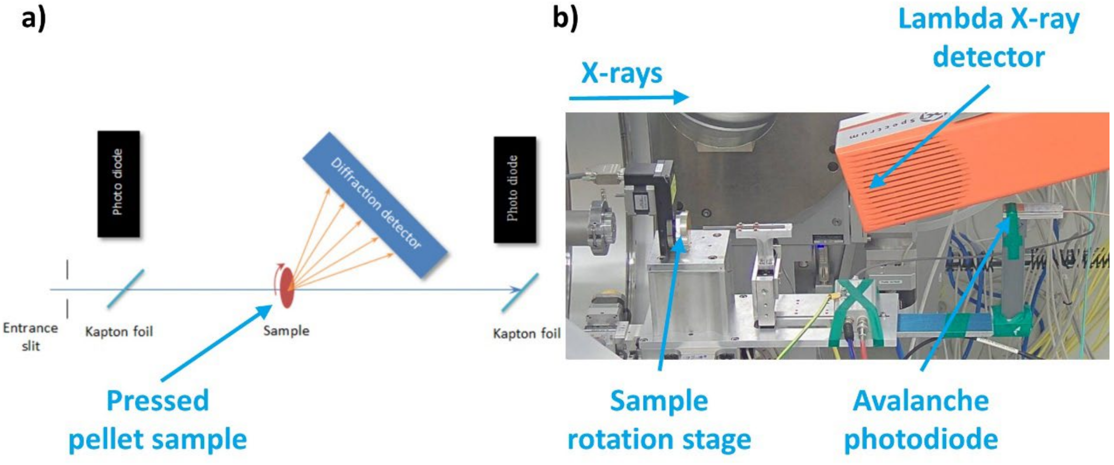

Figure S9. Schematic representation of the experimental set-up used for the XAS and DAFS measurements (a) and photo of the beamline setup (b).

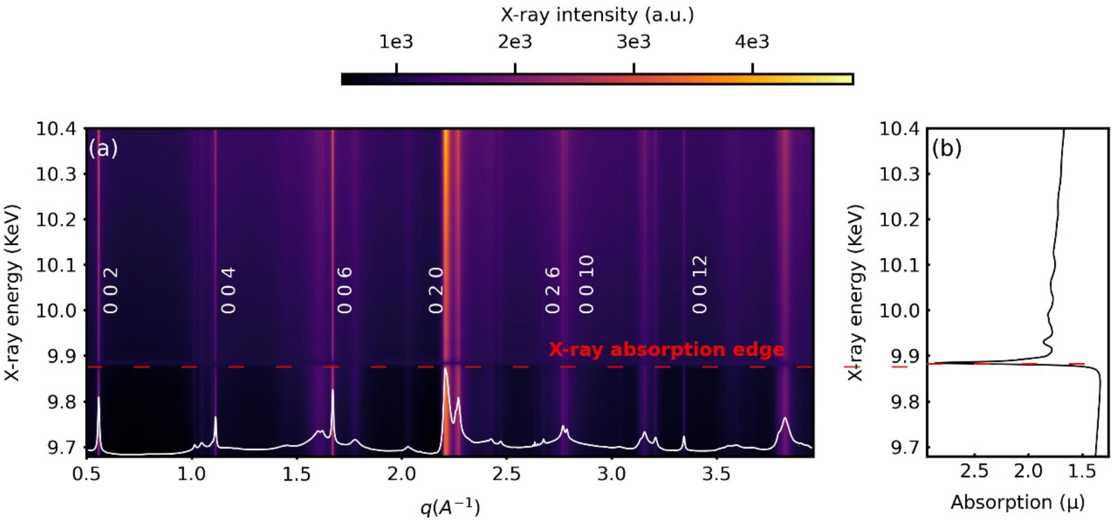

Figure S10. Example of measured DAFS and XAS data of the SmS-TaS_2 sample synthesized at 825 ℃. (a) A 2D plot consisting of 1D powder diffractograms collected at different X-ray energies and stacked along the Y-axis representing the X-ray energy. The white overlay plot at the bottom of the figure represents an example of such a single diffractogram. Several bright lines corresponding to diffraction peaks are indexed. (b) XAS plot where the X-ray energy axis coincides with the same axis of the DAFS data. The red dashed line represents the position of the X-ray absorption edge visible in both DAFS and XAS data.

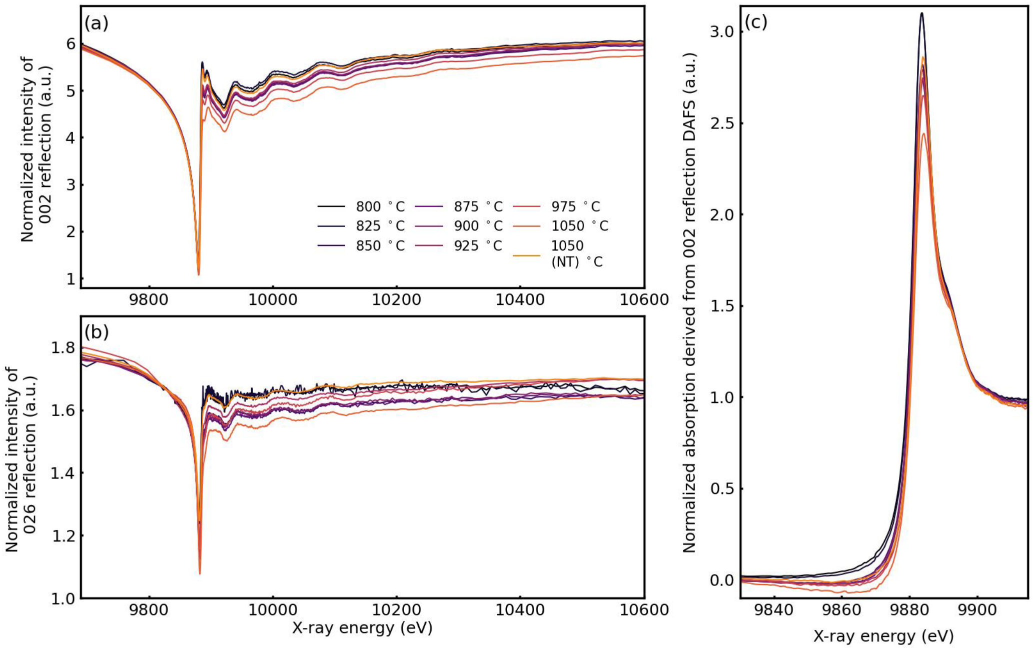

Figure S11. LPA corrected intensity of the 002 (a) and 026 (b) reflections of SmS-TaS_2 samples achieved at different temperatures as a function of X-ray energy; (c) normalized XAS-like spectra derived from the 002 reflection (b) reflection by LDR approach; abnormal behavior of some plots before the edge is due to the LDR approach and normalization artifacts.

Synchrotron-based XRD time series

Figure S12d summarizes the results of a series of XRD experiments in which LaS-TaS_2 powders were prepared at 875 °C (825 °C) for different periods of time. **Figure S12**a presents the XRD pattern of LaS-TaS_2 powders for this series. The preponderance of the 002 peak is indicative of the good crystallinity of the MLC material produced in these syntheses. The 002 peak is common to both (*O-T*) nanotubes and the flakes. Noticeably, after 8 h a small peak assigned to the 004 line of the (*O-T-T*) phase starts to appear. However, after 16 h of reaction time, this peak disappears, and a new peak associated with the 006 line of the (*O-T-T-T*) phase starts to rise and becomes further extenuated after 90 h annealing period. This effect clearly seen in **Figure S12**b showing the evolution of the intensity ratio between the 004/002 peaks of the (*O-T-T*) and (*O-T*) phases respectively. **Figure S12**c shows the intensity ratio between the 006/002 peaks of the (*O-T-T-T*) and (*O-T*) phases of the LaS-TaS_2 MLC with the synthesis time. This surprising result suggests that an auto-catalytic process in which the (*O-T-T*) phase is conductive to the formation of the

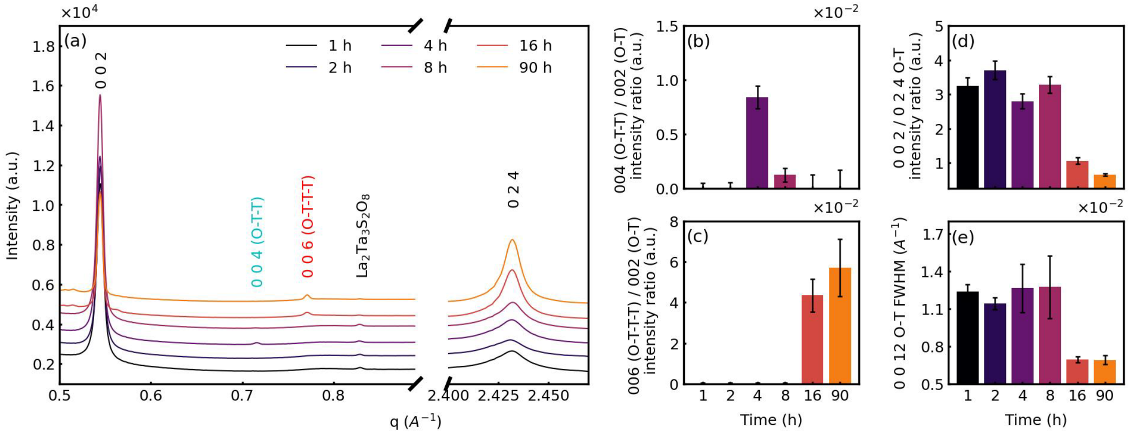

Figure S12. Enlarged areas of the diffractograms of the LaS-TaS_2 samples obtained at 875 °C with varying the reaction time (a), intensity ratios of 002/026 reflections of (*O-T*) phase (b), 002 (*O-T*) diffraction peak FWHM (c), 004 (*O-T-T*) / 002 (*O-T*) (c) and 006 (*O-T-T-T*) / 002 (*O-T*) (d) peak intensity ratios.

(*O-T-T-T*) is operating here. In other words, the deintercalation of a LaS layer with a distorted rocksalt structure from the LaS-TaS_2 superstructure does not occur randomly and is facilitated by an adjacent (*O-T-T*) structure where a prior LaS layer has been deintercalated already. Such auto-catalytic processes are common in intercalation chemistry including Li-ion batteries.[15, 16] The self-catalytic nature of the conversion of the (*O-T-T*) into (*O-T-T-T*) phases become evident from these two block diagrams. While the (*O-T-T*) rises after 4 h reaction time, it disappears and transforms into (*O-T-T-T*) phase after 16 h.

Figure S12d displays the ratio between the XRD reflection peaks 002/024 of the LaS-TaS_2 powders for different reaction times. As pointed out in **Table 1**, while the 002 peak is obtained from the reflections of both nanotubes and platelets, the 024 peak pertains exclusively to the flakes and is forbidden for the nanotubes. Therefore, one can conclude that the concentration of nanotubes increases from 1 to 2 h and then it goes down gradually on the expense of the flakes. A drastic reduction in this ratio occurs, however, after 16 hours and further reduction is observed after 90 h. This picture is corroborated also by the FWHM of the 0012 peak which is almost unchanged during the first 8 h and then it falls indicating the growth of MLC crystallites with large domain-size.

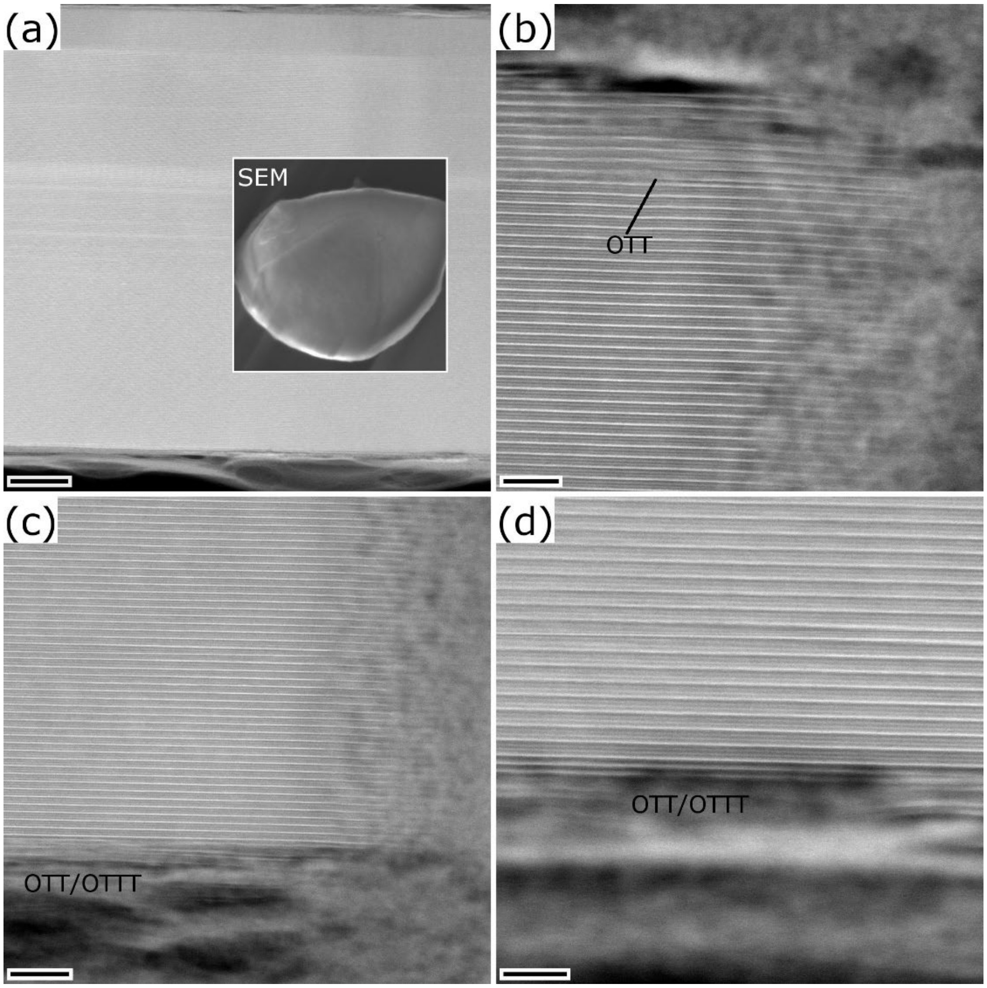

Figure S13. Cross-sectional analysis of a flake of LaS-TaS_2 annealed for 90h at 875ºC. (a) HAADF-STEM image of a cross-section prepared by FIB from a flake (inset SEM image, width of 4 um) with a thickness of 380 nm. (b) The formation of an *O-T-T* stacking via deintercalation of the LaS layer can be observed at the horizontal edge of the flake. (c,d) The formation of considerable amounts of *O-T-T* and *O-T-T-T* is observed at the bottom, vertical edge of the flake, which is found almost over the entire length of the flake. Scale bars are (a) 50 nm, (b) 6 nm, (c) 9 nm and (d) 5 m.

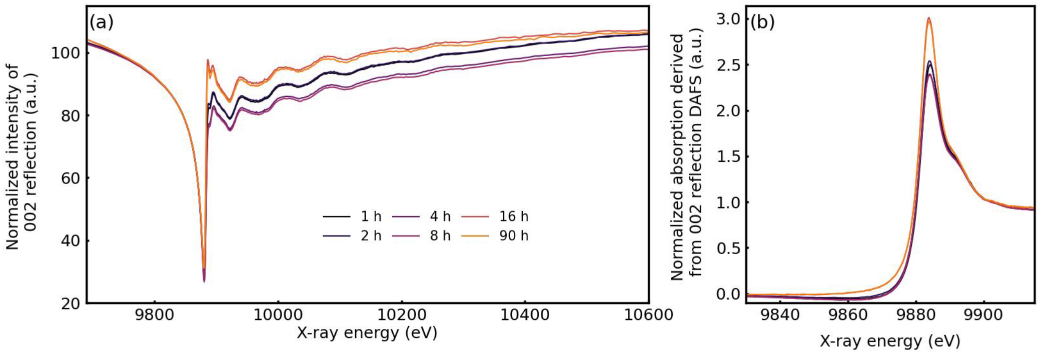

Figure S14. LPA corrected intensity of the 002 (a) reflection of LaS-TaS_2 samples obtained at 875 ℃ with varying reaction time as a function of X-ray energy; (b) normalized XAS-like spectra derived from the 002 (b) reflection by LDR approach; abnormal behavior of some plots before the edge is due to the LDR approach and normalization artifacts.

References

(1) Sreedhara, M. B.; Khadiev, A.; Zheng, K.; Hettler, S.; Serra, M.; Castelli, I. E.; Arenal, R.; Novikov, D.; Tenne, R. Nanotubes from Lanthanide-Based Misfit-Layered Compounds: Understanding the Growth, Thermodynamic, and Kinetic Stability Limits. *Chem. Mater.* **2024**, *36* (9), 4736-4749. DOI: 10.1021/acs.chemmater.4c00481.

(2) Lengeler, B.; Schroer, C.; Tümmler, J.; Benner, B.; Richwin, M.; Snigirev, A.; Snigireva, I.; Drakopoulos, M. Imaging by parabolic refractive lenses in the hard X-ray range. *Journal of Synchrotron Radiation* **1999**, *6* (6), 1153-1167. DOI: 10.1107/S0909049599009747.

(3) Snigirev, A.; Kohn, V.; Snigireva, I.; Lengeler, B. A compound refractive lens for focusing high-energy X-rays. *Nature* **1996**, *384* (6604), 49-51. DOI: 10.1038/384049a0.

(4) Sreedhara, M. B.; Khadiev, A.; Zheng, K.; Hettler, S.; Serra, M.; Castelli, I. E.; Arenal, R.; Novikov, D.; Tenne, R. Nanotubes from Lanthanide-Based Misfit-Layered Compounds: Understanding the Growth, Thermodynamic, and Kinetic Stability Limits. *Chem Mater* **2024**, *36* (9), 4736-4749. DOI: 10.1021/acs.chemmater.4c00481 From NLM PubMed-not-MEDLINE.

(5) Amelinckx, S.; Devouard, B.; Baronnet, A. Geometrical Aspects of the Diffraction Space of Serpentine Rolled Microstructures: their Study by means of Electron Diffraction and Microscopy. *Acta Crystallographica Section A Foundations of Crystallography* **1996**, *52* (6), 850-878. DOI: 10.1107/S0108767396006605.

(6) Zhang, X. B.; Zhang, X. F.; Amelinckx, S.; Van Tendeloo, G.; Van Landuyt, J. The reciprocal space of carbon tubes: a detailed interpretation of the electron diffraction effects. *Ultramicroscopy* **1994**, *54* (2-4), 237-249. DOI: 10.1016/0304-3991(94)90123-6.

(7) Zhang, X. F.; Zhang, X. B.; Van Tendeloo, G.; Amelinckx, S.; Op de Beeck, M.; Van Landuyt, J. Carbon nano-tubes; their formation process and observation by electron microscopy. *Journal of Crystal Growth* **1993**, *130* (3-4), 368-382. DOI: 10.1016/0022-0248(93)90522-X.

(8) Kawaguchi, T.; Fukuda, K.; Matsubara, E. Site- and phase-selective x-ray absorption spectroscopy based on phase-retrieval calculation. *Journal of Physics Condensed Matter* **2017**, *29* (11). DOI: 10.1088/1361-648X/aa53bb.

(9) Kawaguchi, T.; Fukuda, K.; Tokuda, K.; Shimada, K.; Ichitsubo, T.; Oishi, M.; Mizuki, J.; Matsubara, E. Revisit to diffraction anomalous fine structure. *Journal of Synchrotron Radiation* **2014**, *21* (6), 1247-1251. DOI: 10.1107/S1600577514015148.

(10) Kishimoto, S. Behavior of a fast counting system with an avalanche photodiode detector for synchrotron X-rays. *Nuclear Instruments and Methods in Physics Research, Section A:*

Accelerators, Spectrometers, Detectors and Associated Equipment **1997**, *397* (2-3), 343-353. DOI: 10.1016/S0168-9002(97)00816-4.
(11) Baron, A. Q.; Kishimoto, S.; Morse, J.; Rigal, J. M. Silicon avalanche photodiodes for direct detection of X-rays. *J Synchrotron Radiat* **2006**, *13* (Pt 2), 131-142. DOI: 10.1107/S090904950503431X From NLM Medline.
(12) Kieffer, J.; Valls, V.; Blanc, N.; Hennig, C. New tools for calibrating diffraction setups. *Journal of Synchrotron Radiation* **2020**, *27* (2), 558-566. DOI: 10.1107/S1600577520000776.
(13) Newville, M.; Stensitzki, T.; Allen, D. B.; Ingargiola, A. LMFIT: Non-Linear Least-Square Minimization and Curve-Fitting for Python. In *https://dx.doi.org/10.5281/zenodo.11813*, 2014.
(14) Newville, M. Larch: An Analysis Package for XAFS and Related Spectroscopies. *Journal of Physics: Conference Series* **2013**, *430*. DOI: 10.1088/1742-6596/430/1/012007.
(15) Charalambous, H.; Borkiewicz, O. J.; Colclasure, A. M.; Yang, Z.; Dunlop, A. R.; Trask, S. E.; Jansen, A. N.; Bloom, I. D.; Ruett, U.; Wiaderek, K. M.; et al. Comprehensive Insights into Nucleation, Autocatalytic Growth, and Stripping Efficiency for Lithium Plating in Full Cells. *ACS Energy Lett.* **2021**, *6* (10), 3725-3733. DOI: 10.1021/acsenergylett.1c01640.
(16) MacNeil, D. D.; Christensen, L.; Landucci, J.; Paulsen, J. M.; Dahn, J. R. An Autocatalytic Mechanism for the Reaction of $Li_x CoO_2$ in Electrolyte at Elevated Temperature. *J. Electrochem. Soc.* **2000**, *147* (3), 970. DOI: 10.1149/1.1393299.
